# Single-cell transcriptome analysis of the heterogeneous effects of differential expression of tumor PD-L1 on responding TCR-T cells

**DOI:** 10.7150/thno.55075

**Published:** 2021-03-05

**Authors:** Renpeng Ding, Shang Liu, Shanshan Wang, Huanyi Chen, Fei Wang, Qumiao Xu, Linnan Zhu, Xuan Dong, Ying Gu, Xiuqing Zhang, Cheng-Chi Chao, Qianqian Gao

**Affiliations:** 1 BGI-Shenzhen, Shenzhen 518083, China.; 2 BGI Education Center, University of Chinese Academy of Sciences, Shenzhen 518083, China.; 3 Guangdong Provincial Key Laboratory of Human Disease Genomics, Shenzhen Key Laboratory of Genomics.; 4 Guangdong Provincial Key Laboratory of Genome Read and Write, BGI-Shenzhen, Shenzhen 518083, China.; 5 Shenzhen Bay Laboratory, Shenzhen 518132, China.

**Keywords:** TCR-T, PD-L1, differential expression, single-cell RNA sequencing, melanoma

## Abstract

**Rationale:** TCR-T cell therapy plays a critical role in the treatment of malignant cancers. However, it is unclear how TCR-T cells are affected by PD-L1 molecule in the tumor environment. We performed an in-depth evaluation on how differential expressions of tumor PD-L1 can affect the functionality of T cells.

**Methods:** We used MART-1-specific TCR-T cells (TCR-T_MART-1_), stimulated with MART-1_27-35_ peptide-loaded MEL-526 tumor cells, expressing different proportions of PD-L1, to perform cellular assays and high-throughput single-cell RNA sequencing.

**Results:** Different clusters of activated or cytotoxic TCR-T_MART-1_ responded divergently when stimulated with tumor cells expressing different percentages of PD-L1 expression. Compared to control T cells, TCR-T_MART-1_ were more sensitive to exhaustion, and secreted not only pro-inflammatory cytokines but also anti-inflammatory cytokines with increasing proportions of PD-L1^+^ tumor cells. The gene profiles of chemokines were modified by increased expression of tumor PD-L1, which concurrently downregulated pro-inflammatory and anti-inflammatory transcription factors. Furthermore, increased expression of tumor PD-L1 showed distinct effects on different inhibitory checkpoint molecules (ICMs). In addition, there was a limited correlation between the enrichment of cell death signaling in tumor cells and T cells and increased tumor PD-L1 expression.

**Conclusion:** Overall, though the effector functionality of TCR-T cells was suppressed by increased expression percentages of tumor PD-L1 *in vitro*, scRNA-seq profiles revealed that both the anti-inflammatory and pro-inflammatory responses were triggered by a higher expression of tumor PD-L1. This suggests that the sole blockade of tumor PD-L1 might inhibit not only the anti-inflammatory response but also the pro-inflammatory response in the complicated tumor microenvironment. Thus, the outcome of PD-L1 intervention may depend on the final balance among the highly dynamic and heterogeneous immune regulatory circuits.

## Introduction

Programmed cell death-ligand-1 (PD-L1) is the ligand of programmed death-1 (PD-1). They are encoded by *CD274* and *PDCD1*, respectively. PD-L1 is expressed in many cancer tissues such as melanoma 1, a widely recognized immunogenic neoplasm. Expression of PD-L1 is undetectable in most normal tissues, but can be induced by inflammatory cytokines, especially interferon-γ (IFN-γ) in various cell types 2-4. As a strategy to evade immune responses and impair T cell response, PD-L1 is often up-regulated on tumor cells and induces T cell anergy, exhaustion, or apoptosis upon engagement with PD-1 expressed on tumor infiltrating lymphocytes (TILs) 1, 5. Expression of PD-L1 is not restricted to tumor cells; PD-L1 is also expressed on TILs and this expression correlates with aggressive tumors, demonstrating the immunosuppressive role of PD-L1 6, 7. Binding of PD-1 and PD-L1 impairs T cell activation by interfering with the Ras-Raf-MEK-ERK and PI3K-AKT signaling pathways that promote T cell proliferation and differentiation 8. In addition to binding PD-1, PD-L1 has been reported to interact with CD80 in *cis* to modulate T cell function and the tumor microenvironment 9, 10.

The PD-1/PD-L1 signaling pathway plays an important role in tumor evasion from host immune responses 11. Inhibitors of PD-1 and PD-L1 have been studied in various tumor types and have now been approved for treating many malignancies, including melanoma, non-small-cell lung cancer (NSCLC), and bladder cancer 12-16. PD-L1 expression on tumor cells and tumor infiltrating antigen presenting cells (APCs) has been approved as a companion biomarker to aid clinicians in determining whether to use these inhibitors as therapies 17-22. With these treatments, a positive correlation between higher levels of PD-L1 expression and higher response rates in melanoma has also been demonstrated 23-25. Yet, some studies show that PD-L1 expression is insufficient in predicting a beneficial response from immune checkpoint inhibitor (CPI) therapy and PD-L1 expression level alone is a poor predictive biomarker of overall survival 26, 27.

While the role of PD-L1 has been studied in many literatures 4-6, 22, 28, elucidating the exact relationship between PD-L1 expression and T cell function in bulk T-cell populations has remained difficult. Furthermore, while T cell receptor-engineered T (TCR-T) cell therapy has shown promising results in treating solid tumors 29, few studies have systemically investigated how tumor PD-L1 can affect the functionality of tumor antigen-specific TCR-T cells. Thus, it is important to understand how PD-L1 and its expression levels on tumor cells at a single-cell level affects the efficacy of T-cell immunotherapy.

In this study, we conducted multiplex cytokine and cell cytotoxicity assays in conjugation with high-throughput single-cell mRNA sequencing (scRNA-seq) to investigate the immunoregulatory effect of tumor PD-L1 on responding TCR-T cells. Increased expression of tumor PD-L1 suppressed cell cytotoxicity and cytokine secretion of TCR-T cells *in vitro*, while the diverse capabilities of scRNA transcriptome analysis revealed that both anti-inflammatory and pro-inflammatory responses were triggered by the increased expression of tumor PD-L1, providing possible explanations for the contradicting results of using tumor PD-L1 expression to predict clinical efficacy of anti-PD-1/PD-L1 therapies. Our research is the first at the single-cell level to analyze the transcriptional features, as well as the cytokine and cytotoxic signatures, of antigen-specific TCR-T cells responding to different tumor PD-L1 ratios.

## Methods

### Cell lines and cell culture

HEK293T (ATCC, CRL-11268) and T2 (174×CEM.T2, CRL-1992) cell lines were purchased from ATCC, and MEL-526 (BNCC340404) cell line was purchased from BNCC. HEK293T and MEL-526 cells were cultured in DMEM (Gibco, 21063029) supplemented with 10% fetal bovine serum (Hyclone, SH30084.03HI), penicillin (100 IU/mL), and streptomycin (50 μg/mL). T2 cells were cultured in IMDM (Gibco, 12440053) supplemented with 20% fetal bovine serum (Hyclone, SH30084.03HI), penicillin (100 IU/mL), and streptomycin (50 μg/mL). CD8^+^ T cells were cultured in HIPP-T009 (Bioengine, RG0101302) supplemented with 2% fetal bovine serum (Hyclone, SH30084.03HI) containing IL-2 (20 ng/ml), IL-7 (10 ng/ml), and IL-15 (10 ng/ml).

### Plasmid construction

TCR_MART-1_ sequence was identified from our previous work (data unpublished), and its constant regions were replaced by mouse TCR constant regions α and β, respectively, to prevent mispairing with endogenous TCR. TCR α chains and β chains were linked by a P2A self-cleaving peptide. The recombinant DNA encoding TCR_MART-1_ was synthesized by GeneScript (Nanjing, China) and ligated into pRRLSIN.cPPT.PGK vector (Addgene, 12252).

PD-L1 cDNA ORF Clone in Cloning Vector was purchased from Sino Biological (HG10084). PD-L1 cDNA was cloned into pRRLSIN.cPPT.PGK vector (Addgene, 12252) with ClonExpress II One Step Cloning Kit (Vazyme, C112) according to the user manual.

### Lentivirus production

293T cells were transfected with a mixture of plasmids and packaging constructs of interest (PsPAX2 and PMD2G), as previously described 30. The culture supernatants were collected 72 h after transfection and filtered through a 0.45 µM filter. Subsequently, the supernatants were concentrated by ultracentrifugation at 35,000 rpm for 90 min. The pellet was suspended and stored at -80 °C.

### Generation of tumor cells expressing PD-L1

After lentivirus infection of PD-L1 lentivirus into MEL-526 cells for 2 days, PD-L1^+^ cells were sorted by FACS. Different proportions of PD-L1^+^ tumor cells were obtained by mixing wildtype and PD-L1^+^ MEL-526 cells.

### Generation of MART-1-specific T cells

Human Peripheral Blood Mononuclear Cells (PBMCs) were isolated from the blood of HLA-A*0201-restricted healthy donors with informed consent. CD8^+^ T cells were purified from PBMC via human CD8 MicroBeads (Miltenyi Biotec, 130-045-201) and activated with T Cell TransAct (Miltenyi Biotec, 130-111-160). After 36-48 h, CD8^+^ T cells were transduced with TCR_MART-1_ lentivirus at MOI = 25 in a 6-well or 12-well plate. Simultaneously, polybrene was added to the culture at a final concentration of 2 μg/ml to promote infection efficiency. The well plate was then centrifuged at 800 g at room temperature for 30 min.

### Peptide synthesis

MART-1 originated peptide ELAGIGILTV (HLA-A*0201) was synthesized by GenScript (Nanjing, China) with a purity of ≥ 99.0%. Peptides were dissolved with 100% dimethyl sulfoxide (DMSO; Sigma-Aldrich, D5879-500ML) to 10 mg/ml, and were stored at -20 °C.

### TCR-T cell stimulation with target tumor cell

TCR-T cells and MEL-526 cells (5×10^5 cells/ml concentration, in 200 μl), either pulsed with peptide (final concentration 10 μg/mL) or not, were incubated for 24 h in a round bottom 96-well plate. Afterwards, the co-culture was subjected to scRNA-seq. Unstimulated TCR-T cells (5×10^5 cells/ml) were incubated for 6 h alone before being subjected to scRNA-seq.

### Intracellular staining

Cells were perforated and fixed using Cytofix/Cytoperm kit (BD Pharmingen, 554715). The following antibodies were used: Allophycocyanin (APC)-anti-HLA-A2 antibody (eBioscience, 17-9876-42), Phycoerythrin (PE)-anti-human CD8a antibody (eBioscience, 12-0086-42), APC-anti-human CD274 (PD-L1) antibody (BD Pharmingen, 563741), PE-anti-human CD279 (PD-1) antibody (Biolegend, 367404), PE anti-mouse TCR β chain Antibody (Biolegend, 109207), APC anti-human IFN γ (eBioscience, 502512), PE-anti-human Granzyme B (BD Pharmingen, 561142), APC anti-human CD107a (Biolegend, 328620), PE-anti-Ki67 antibody (Abcam, ab270650).

### Detection and quantification of surface and mRNA expression of PD-L1

PD-L1 expression on T cells was quantified at both protein and RNA levels. For the detection of surface PD-L1 molecules, T_null_ and TCR-T_MART-1_ were co-cultured with MEL-526 cells at an effector-to-target ratio of 1:1 for 24 h. The mixed cells were subsequently harvested and stained with anti-mouse TCR β chain-PE, anti-CD3-FITC, and anti-PD-L1-APC antibodies, and then detected by BD FACS AriaIII. For the mRNA quantification, *CD274* in each cell was normalized and calculated according to the data generated from the scRNA sequencing. T cells containing reads that mapped to the *CD274* locus were considered as PD-L1 positive cells.

### Cell cytotoxicity assays

Target cells were labeled with Carboxyfluorescein succinimidyl ester (CFSE; Invitrogen) and co-cultured with 50% TCR-T cells at an E:T ratio of 1:2. After 24 h, cells were collected and stained with PI and subsequently detected by FACS.

### Cytokine secretion measurement

The secretion of TNF-α, granzyme A, and granzyme B by T cell were evaluated using a BDTM cytometric bead array (CBA) system. T_null_ or TCR-T_MART-1_ cells were co-cultured with MEL-526 cells either pulsed with or without peptide and supernatants were collected 24 h later. CBA assay was performed according to the instruction manual.

### Statistical analysis

Data analysis was performed using PRISM 6 (GraphPad Software) and RStudio. *P < 0.05, **P < 0.01, ***P < 0.001. Values are presented as mean standard deviation (SD). Error bars represent the SD.

### ScRNA-seq

Single-cell 3' mRNA transcriptome profiling was performed using a negative pressure orchestrated DNBelab C4 system according to the workflow 31.

### ScRNA-seq data preprocessing

For all the samples, the iDrop Software Suite (v.1.0.0) was used to perform sample de-multiplexing, barcode processing, and single-cell 3' unique molecular identifier (UMI) counting with default parameters. Processed reads were then aligned onto the complete UCSC hg38 human genome by splicing-aware aligner STAR with default parameters. Valid cells were automatically identified based on the UMI number distribution of each cell. The following filtering criteria was used to obtain high-quality single cells: the number of genes in each cell in the range of 400 to 6000, the ratio of mitochondrial genes less than 0.05, and the number of UMI greater than 1000.

### Unsupervised clustering

The expression matrix obtained in the above steps was used as input to Seurat v. 3 to perform batch effect correction, standardization, dimensionality reduction, and clustering. First, the “LogNormalize” function was applied to normalize the data. Next, the “vst” method in the “FindVariableFeatures” function was used to detect variable genes, and the top 3000 variable genes were selected for downstream analysis. Then, the “FindIntegrationAnchors” and “IntegrateData” functions were used to correct batch effects. Finally, the top 3000 variable genes were applied for PCA dimensionality reduction. The UMAP was performed on the top 20 principal components for visualizing these cells. At the same time, graph-based clustering was performed on the PCA-reduced data for clustering analysis with Seurat v.3. The resolution was set to one to obtain the most representative result.

### Differential gene expression analysis

We applied the FindMarkers to differential gene expression analysis. For each cluster of T cells and tumor cells, DEGs were generated relative to all of the other cells. A gene was considered significant with adjusted P < 0.05 and logFC > 0.25. To compare DEGs across CD8+ T cells and tumor cells under different experimental conditions, the limma method was used with the parameters recommended in the user guide for analysis. DEGs were identified when they met the following criteria: FDR adjusted p value of F test < 0.01.

### Developmental trajectory inference

The Monocle (version 2) algorithm with the signature genes of different functional clusters was applied to order CD8^+^ T cells excluding clusters expressing proliferating or mitochondrial genes in pseudo time. UMI value was first converted into normalized mRNA counts by the “relative2abs” function in monocle and created an object with parameter “expressionFamily = negbinomial.size” according to the Monocle tutorial. The batch effect was regressed out using the “reduceDimension” function with default parameters. The CD8^+^ T cell differentiation trajectory was determined by the default parameters of Monocle.

Next, the BEAM function was used to detect genes which separate cells into the calculated states. We used the plot_multiple_branches_heatmap function to separate the above gene set with a q-value less than or equal to 10e-300 with hierarchical clustering using num_clusters = 3 and“branches” set to the terminal branchpoints for each respective sample.

### Gene set enrichment analysis

Gene Ontology (GO) enrichment analysis was performed on the differential genes of each cluster, and the results were used for cell type definition. The “enrichGO” function in the “clusterProfiler” package was used to perform GO analysis using the corresponding default parameters. Pathways with the q value < 0.05 corrected by FDR were used for analysis.

### GSVA

GSVA was used to identify the molecular phenotype of each cluster with the normalized UMI data. The average normalized expression across T cell clusters was first obtained. Then, GSVA scores of gene sets for different clusters were calculated. GSVA values were plotted as a heatmap using R package “pheatmap”.

### Data sets and processing

The expression profiles and clinical information of human metastatic melanoma was downloaded from GEO website (GSE78220). The patients on-treatment or with previous MAPKi treatment were discarded. These samples were split into two groups (PDL1^high^ and PDL1^low^) according to the expression levels of *CD274* in tumor cells (cutoff = 0.003). To correct for the effect of cancer cell levels within each sample, the expression of *CD274* in tumor was divided by that of the geometric mean of cancer marker genes (HSPB1 and KRT18). The cutoff of relative expression was determined by the distribution of the relative expression and median value. The samples with relative expression below the cutoff were categorized as the PDL1^low^ group and those with relative expression above the cutoff were classified as the PDL1^high^ group. Next, the relative expression of selected genes in CD8^+^ T cell was calculated as above. The expression of selected genes was divided by the geometric mean of CD8^+^ T cell marker genes (CD3D, CD3E, CD3G, CD8A and CD8B) for the effect of different levels of tumor PD-L1. The relative expression of selected genes was compared between PDL1^low^ and PDL1^high^ groups. The p value was determined by Permutation test.

### Data availability

The data that support the findings of this study have been deposited into CNGB Sequence Archive (CNSA: https://db.cngb.org/cnsa/) of CNGBdb with accession number CNP0001109.

### Ethics approval and consent to participate

The study was approved by the Institutional Review Board on Bioethics and Biosafety of BGI. A written informed consent was regularly obtained from all donors.

## Results

### Increased tumor PD-L1 expression suppressed cytotoxicity and cytokine secretion of TCR-T_MART-1_

**Figure 1:**
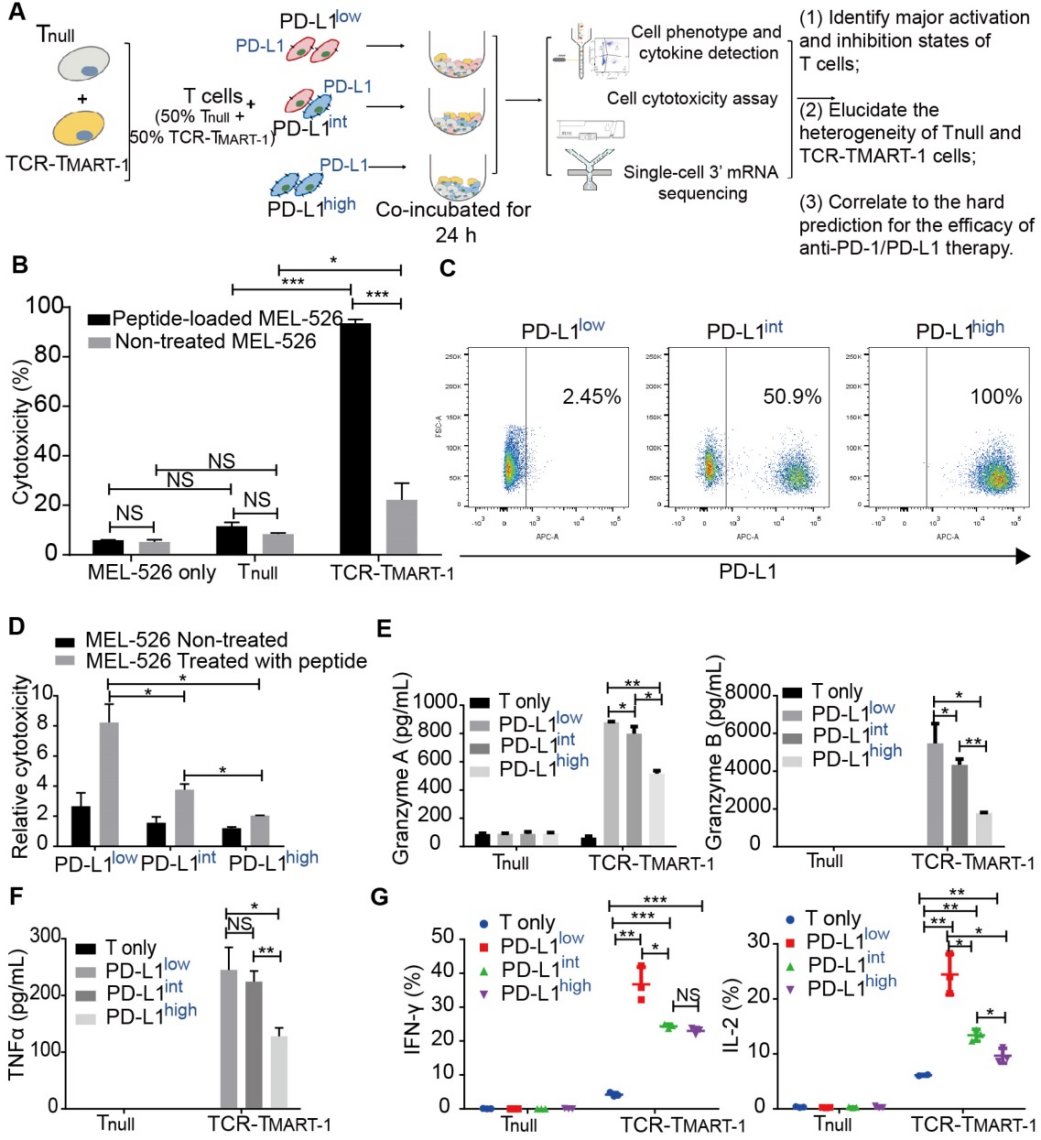
** PD-L1 expression on melanoma MEL-526 cells pulsed with MART-1_26-35_ peptide inhibited cytotoxicity and cytokine secretion of TCR-T_MART-1_. (A)** Overview of the study design. T_null_, control T cells; TCR-T_MART-1_, MART-1 specific TCR-T cells. **(B)** TCR-T_MART-1_ cytotoxicity against MEL-526 cells loaded with or without MART-1_26-35_ peptide at an E:T ratio of 1:1. **(C)** Flow cytometric analysis of PD-L1 expression on PD-L1^low^, PD-L1^int^, and PD-L1^high^ MEL-526 cells. **(D)** TCR-T_MART-1_ cytotoxicity was inhibited by tumor PD-L1 in a dose dependent manner. T and TCR-T cells were incubated with different proportions of PD-L1^+^ MEL-526 cells for 24 h. **(E)** Secretion of Granzyme A and Granzyme B by TCR-T_MART-1_ was inhibited by increased tumor PD-L1. T_null_ and TCR-T_MART-1_ were co-cultured with MART-1_26-35_ peptide loaded-MEL526 cells with different proportions of PD-L1 expression at an E:T ratio of 1:1, and the secretion was detected by the Cytometric Bead Array (CBA) system. **(F)** Secretion of TNF-α by TCR-T_MART-1_ was inhibited by an increased proportion of PD-L1 expression among MEL-526 cells. **(G)** Secretion of IFN-γ and IL-2 by TCR-T_MART-1_ was inhibited by an increased percentage of PD-L1 expression among MEL-526 cells. Error bars represent S.E.M. (N = 3). (∗) 0.01 < P < 0.05, (∗∗) 0.001 < P < 0.01, (∗∗∗) P < 0.001. NS, not significant. N = 3.

Cytotoxicity and cytokine secretion assays, along with scRNA-seq, were conducted to evaluate TCR-T cells stimulated by MEL-526 melanoma cells with different proportions of PD-L1 expression (Figure 1A). This approach made it possible to quantitatively analyze the T-cell activation state in relation to their subtypes and gene expression. HLA-A*0201/Melan-A-specific TCR sequence (designated as TCR_MART-1_) was obtained from T cells stimulated with Melan-A (aa27-35, LAGIGILTV) peptide (data unpublished). Melan-A, also known as MART-1, is a melanocytic marker 32. Human TCRα and TCRβ sequences fused with the murine TCR constant region were synthesized and cloned into a lentiviral vector (Figure S1A). T cells that either expressed or did not express TCR_MART-1_ were designated as TCR-T_MART-1_ and T_null_, respectively. After lentiviral transduction into CD8^+^ T cells, 17.5% of T cells was TCR-T_MART-1_, which reached 97.2% after fluorescence-activated cell sorting (FACS) (Figure S1B). To verify the cytolytic capacity 33-35, TCR-T_MART-1_ were stimulated by peptide-loaded MEL-526 cells or a mock control at an effector-to-target (E:T) ratio of 1:1. Compared to T_null_, TCR-T_MART-1_ killed MEL-526 cells more efficiently when MEL-526 cells were loaded with the MART-1_27-35_ peptide (Figure 1B). TCR-T_MART-1_ similarly killed T2 cells, another target cell line (Figure S1C).

To investigate the immunosuppressive role of tumor PD-L1, PD-L1 was overexpressed (OE) on MEL-526 cells (Figure S1D). Different percentages of PD-L1^+^ tumor cells were obtained by mixing OE with wild-type (WT) MEL-526 cells based on the clinical PD-L1 expression ratio 36, 37. Three tumor cell populations with different percentages of MEL-526 expressing PD-L1 were used in the study: PD-L1^low^ (without exogenous PD-L1, 2.45%), PD-L1^int^ (intermediate, 50.9%), and PD-L1^high^ (high, 100%) (Figure 1C). The cytolytic activity of TCR-T_MART-1_ was inhibited by increasing the percentage of tumor cells expressing PD-L1 (Figure 1D), affecting the secretion of Granzymes (Figure 1E) and pro-inflammatory cytokines in T_null_ and TCR-T_MART-1_, including TNFα (Figure 1F), IFNγ, and IL2 (Figure 1G).

### Distinct clusters of different cell subpopulations were identified by single-cell transcriptome analysis

**Figure 2:**
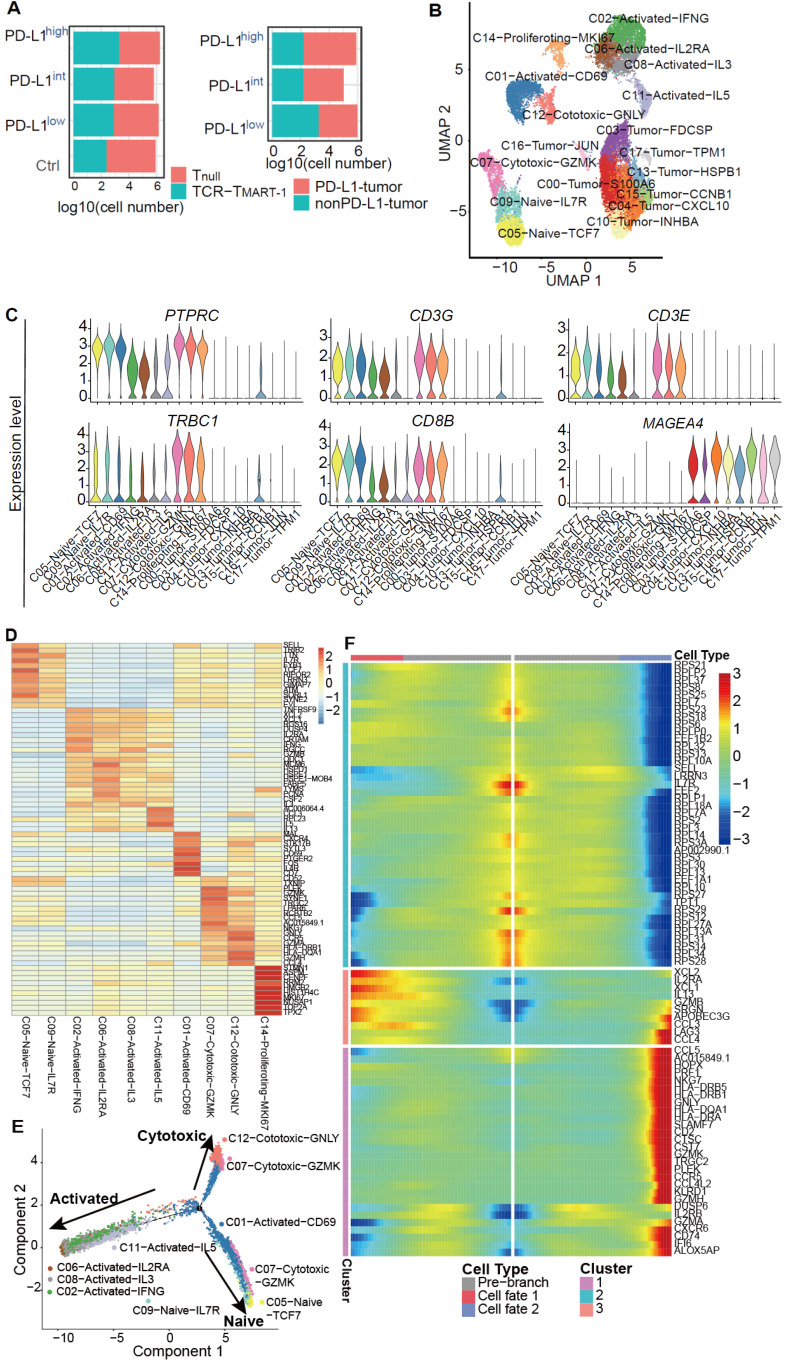
Single-cell level analysis revealed distinct cell subpopulations.** (A)** Cell number of T_null_, TCR-T_MART-1_, MEL-526 (nonPD-L1), and MEL-526 (PD-L1 OE) of four experiment groups. **(B)** The UMAP projection of T cells and tumor cells, showing 18 main clusters in different colors. The phenotype description of each cluster is determined by marker gene expression of T cells and tumor cells. **(C)** Violin plots showing the expression profile of marker genes of T cells and tumor cells in the 18 clusters.** (D)** Heatmap of T cell clusters with unique signature genes.** (E)** The ordering of T cells along pseudotime in a two-dimensional state-space defined by Monocle2. Cell orders were inferred from the expression of most dispersed genes across T cell populations. Each point corresponds to a single cell, and each color represents a T cell cluster.** (F)** Heat map showing the gene expression that separated cells into the specialized states detected by BEAM.

To investigate the effect of increased percentages of tumor PD-L1 on gene expression, single-cell transcriptome profiling was performed using a negative pressure orchestrated DNBelab C4 system 31. Transcriptome profiling of a total of 20,888 cells from four conditions was obtained after filtering out cells of low quality (Figure 2A). To investigate the intrinsic T cell heterogeneity, unsupervised clustering was performed (Figure 2B) after adjusting for the batch effect between these groups (Figure S2A). T and tumor cells were identified by the expression of classic cell type markers, including *PTPRC*, *CD3G*, *CD3E*, *TRBC1*, and* CD8B* for T cells and *MAGEA 4* for MEL-526 cells (Figure 2C). Based on the expression of signature genes, T cells were composed of clusters 1, 2, 5, 6, 7, 8, 9, 11, 12, and 14 (Figure 2B). Exogenous TCR_MART-1_ was detected in clusters 1, 8, 12, 2, 6, and 11, with especially strong signals in the last three clusters (Figure S2B). Furthermore, differentially expressed genes (DEGs) and known functional markers indicated clusters of naïve, activated, cytotoxic, and proliferating CD8^+^ T cells (Figure 2B, 2D). Tumor cell clusters 0, 3, 4, 10, 13, 15, 16, and 17 were further identified as well (Figure S2C).

To understand T cell state transitions, an unsupervised inference method Monocle 2 38 was applied to construct the potential development trajectories of nine T cell clusters (cluster 14 was excluded due to its distinct expression of *MKI67*). Cells from all clusters were aggregated according to expression similarities to form a relative process in pseudotime (Figure S2D), which began with clusters 5 and 9 naïve cells, followed by C07-Cytotoxic-GZMK and C01-Activated-CD69 (Figure 2E). C06, C08, C02, and C11 activated cells were located in opposite directions with C12 and C07 cytotoxic cells in the pseudotime trajectory plot, demonstrating the diverse functions of these cells. Continuous transcriptome changes following the two different differentiation directions were analyzed (Figure 2F). The expression of *XCL2*,* IL2RA*, *XCL1*, *IL13*, and *GZMB* etc. was increased when differentiating towards the cell-activated direction (cell fate 1), while the expression of *CCL5*, *NKG7*, *GNLY*, *CST7*, and *GZMK* etc. was upregulated when following the cell-cytotoxic direction (cell fate 2) (Figure 2F). In addition, the expression of genes encoding ribosome proteins was downregulated in both directions, as were the naïve marker genes *SELL* and *IL7R* (Figure 2F). Taken together, while T cells differentiated into activated and cytotoxic populations, the expression of genes related to T cell activation and cytotoxicity was upregulated respectively, and the expression of naïve marker genes was downregulated, reflecting the differentiation directions.

### T cell clusters responded divergently to the increased expression of tumor PD-L1

**Figure 3:**
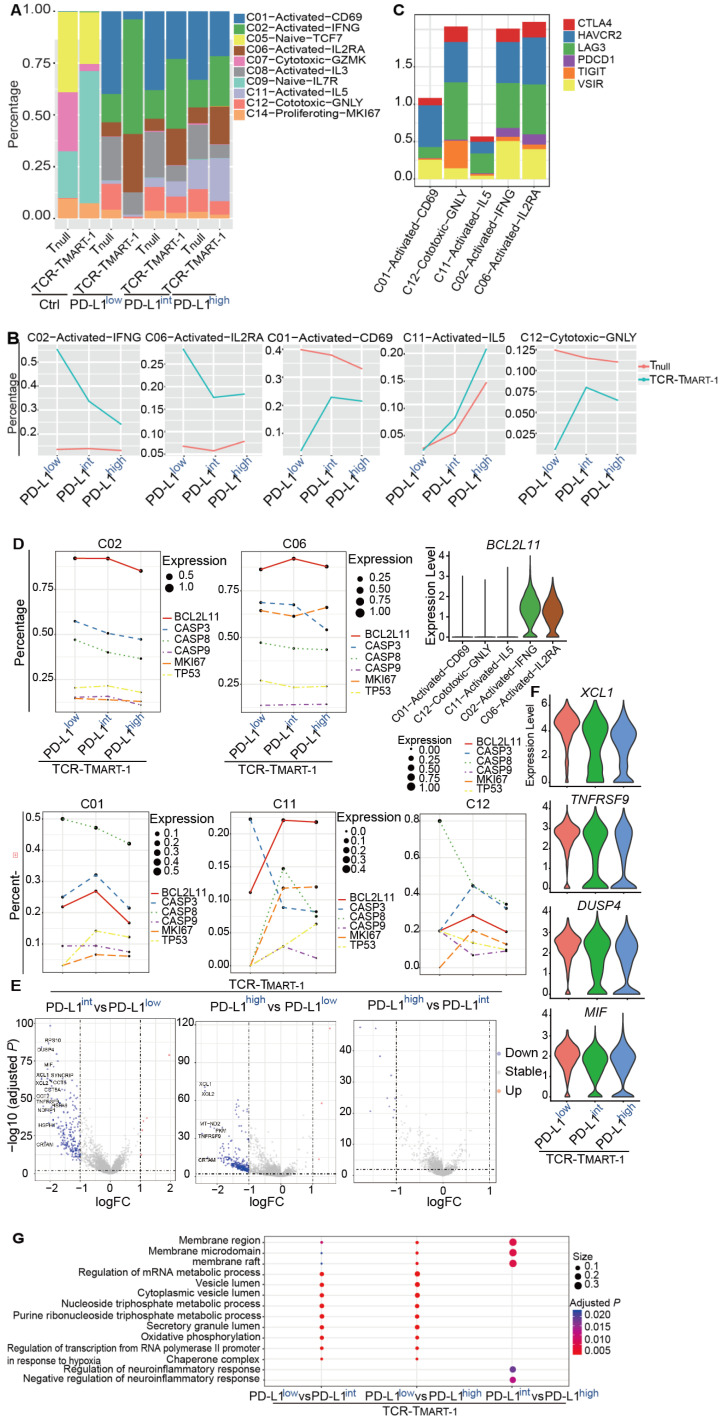
Various responses between T cell clusters, and T_null_ and TCR-T_MART-1_ to different levels of tumor PD-L1. **(A)** Cluster composition of T_null_ and TCR-T_MART-1_. **(B)** The proportion distribution of T cell clusters with the increased tumor PD-L1. **(C)** The bar plot shows the proportion distribution of cells expressing *CTLA4*, *HAVCR2* (*TIM3*), *LAG3*, *PDCD1*, *TIGIT*, and *VSIR* among the five T cell clusters, respectively (cutoff: UMI of the gene > 0). **(D)** The bubble plot shows the proportion distribution of T cells expressing *BCL2L11*, *CASP3*, *CASP8*, *CASP9*, *MKI67*, and *TP53* in TCR-T_MART-1_ responding to differential proportions of PD-L1^+^ tumor, among the five clusters respectively. The size of the point shows the mean expression of genes in the corresponding T cell population. The violin shows the expression distribution of *BCL2L11* among the five clusters. **(E)** Differentially expressed genes in TCR-T_MART-1_ responding to differential proportions of PD-L1^+^ tumor. **(F)** The expression distribution of *XCL1*, *TNFRSF9*, *DUSP4*, and *MIF* in TCR-T_MART-1_ responding to differential proportions of PD-L1^+^ tumor. **(G)** Bubble plot showing the top 10 pathways in T_null_ (left) and TCR-T_MART-1_ (right) compared to the control group, respectively. The color represents pvalue and the size represents gene ratio.

To reveal the structure of the overall T cell population, T cells were classified into T_null_ and TCR-T_MART-1_, and their cluster compositions were investigated. Cluster composition of the control (Ctrl) group was different than that of groups stimulated by tumor cells (Figure 3A). After stimulation, the percentage of C02-Activated-IFNG and C06-Activated-IL2RA of TCR-T_MART-1_ was downregulated, while the fragment of C01-Activated-CD69, C11-Activated-IL15, and C12-Cytotoxic-GNLY was upregulated with increased tumor PD-L1 levels (Figure 3B). Though these cell clusters were all activated or cytotoxic, their percentages were paradoxically modified upon stimulation with tumor cells expressing different ratios of PD-L1. Since not all the activated or cytotoxic T cells were suppressed by a high expression of tumor PD-L1, blocking PD-L1 might downregulate some anti-tumor T cell subpopulation contributing to the complicated possibilities. On the other hand, clusters of T_null_ were not dramatically changed compared to TCR-T_MART-1_ clusters, except C11 which was upregulated with increased tumor PD-L1 (Figure 3B). These results implied that TCR-T_MART-1_ was more sensitive than T_null_ to increasing levels of tumor PD-L1.

Next, the heterogeneity of these five clusters was examined and the top 10 DEGs found were quite distinct (Figure S3A). In addition, cell cytokines, including *IFNG*, *IL2*, and *TNFA*, or cytotoxic genes such as *GNLY*, *PRF1*, *GZMA*, and *GZMB* were not found regularly expressed in these clusters (Figure S3B). Similar to the exhaustion markers, expression of *PDCD1* was rarely found in C01, C12, or C11, but was uniquely detected in C02 and C06, the percentages of which were decreased with higher expression of tumor PD-L1 (Figure 3C). Furthermore, higher expression of *VSIR* was discovered in C02 and C06 as well (Figure 3C), indicating clusters with higher expression of *PDCD1* and *VSIR* may tend to be downregulated by increased expression levels of tumor PD-L1.

Changes in percentages of different T cell clusters with increased tumor PD-L1 might be caused by reduced proliferation or elevated cell death. To clarify the role of cell proliferation and cell death in this process, the expression of the key genes associated with increased tumor PD-L1 was analyzed. Expression of proliferation gene *MKI67* and apoptotic genes, including *TP53*, *BCL2L11*, *CASP3*, *CASP 9*, and *CASP 8* was not dramatically changed in C02 and C06 (Figure 3D), demonstrating that the downregulation of C02 and C06 might not be due to the reduced proliferation or upregulated cell apoptosis. However, C02 and C06 continually expressed higher levels of *BCL2L11* compared to C01, C11, and C12 (Figure 3D). Moreover, the expression of *MKI67* increased with higher levels of tumor PD-L1 in C01, C11, and C12, while the expression of certain apoptotic genes, such as *CASP8* in C01 and C12, and *CASP3* in C11, was downregulated (Figure 3D). Thus, the increased percentages of C01, C11, and C12 with increasing tumor PD-L1 levels might be caused by upregulated cell proliferation and reduced cell death.

In addition to T cell clusters, T_null_ and TCR-T_MART-1_ responded differently to increased levels of tumor PD-L1. TCR-T_MART-1_ was affected more than T_null_ by increased tumor PD-L1 levels (Figure 3E, S3C), and TCR-T_MART-1_ targeting PD-L1^low^ tumors expressed higher levels of not only activation genes, such as *XCL* and *TNFRSF9*, but also anti-inflammatory genes, such as *DUSP4* 39 and *MIF* 40, compared to the TCR-T_MART-1_ targeting PD-L1^int^ and PD-L1^high^ tumors (Figure 3E, 3F). Enriched signaling pathways were then analyzed. Different signaling pathways were enriched in T_null_ and TCR-T_MART-1_ after encountering tumor cells (Figure S3D). Compared to TCR-T_MART-1_ targeting PD-L1^int^ and PD-L1^high^, TCR-T_MART-1_ targeting PD-L1^low^ enriched metabolic and vesicle lumen related signaling pathways (Figure 3G). However, distinct pathways including the membrane region, membrane microdomain, and raft were enriched in TCR-T_MART-1_ targeting PD-L1^int^ compared to that targeting PD-L1^high^ (Figure 3G), though there was no clear evidence as to why these signaling pathways were most affected by different percentages of tumor PD-L1.

### Differential PD-L1 expression heterogeneously regulated the genes associated with TCR-T_MART-1_ functionality

**Figure 4:**
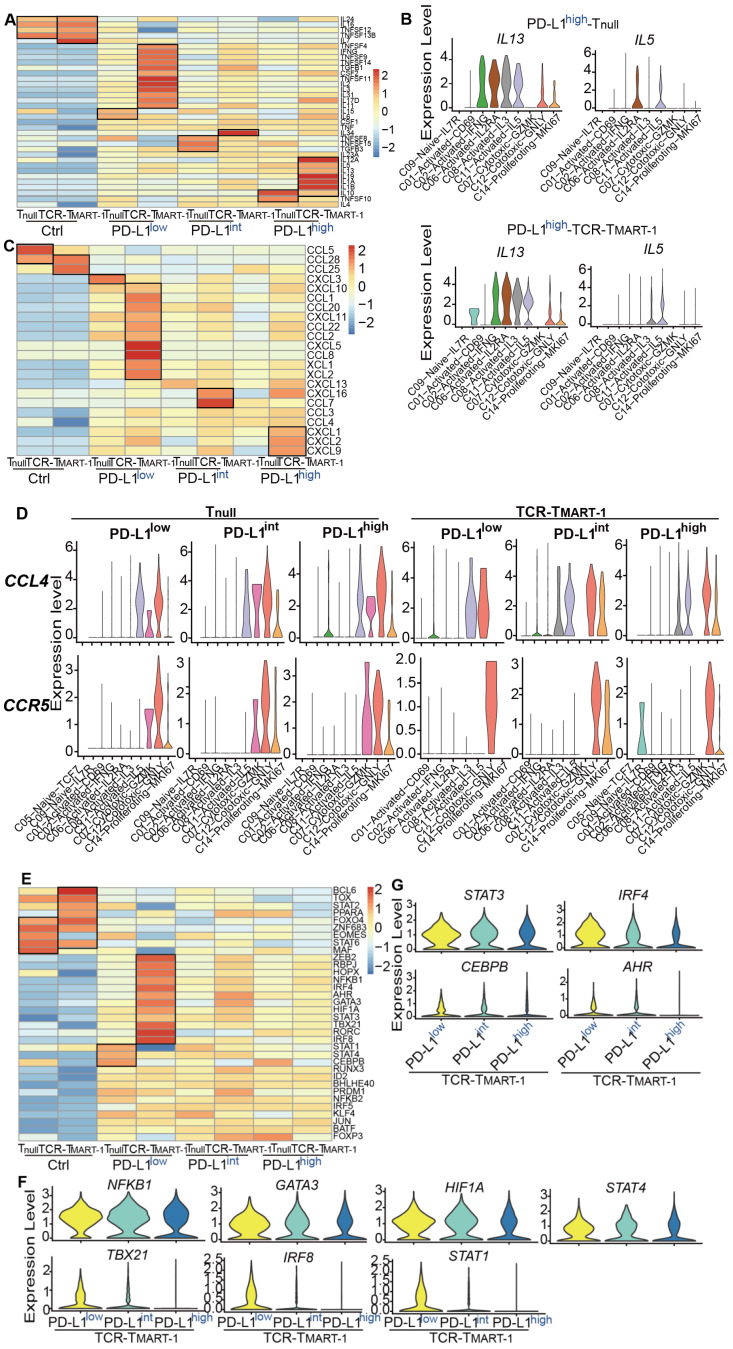
Influences of increased tumor PD-L1 on cellular and molecular responses of T cells. **(A)** The expression profile of cytokines in T_null_ and TCR-T_MART-1_. **(B)** The expression distribution of *IL13* and *IL5* in T_null_ and TCR-T_MART-1_ responding to PD-L1^high^. **(C)** The expression profile of chemokines in T_null_ and TCR-T_MART-1_. **(D)** The expression distribution of *CCL4* and *CCR5* in T cell clusters of T_null_ and TCR-T_MART-1_ responding to differential proportions of PD-L1^+^ tumor. **(E)** The expression profile of transcription factors in T_null_ and TCR-T_MART-1_. **(F)** The expression distribution of *NKFB1*, *GATA3*, *HIF1A*, *STAT4*, *TBX21*, *IRF8*, and *STAT1* in TCR-T_MART-1_ responding to differential proportions of PD-L1^+^ tumor. **(G)** The violin showing the expression levels of *STAT3*, *IRF4*, *CEBPB*, and *AHR* in TCR-T_MART-1_ responding to differential proportions of PD-L1^+^ tumor.

The expression of cytokines, chemokines, and transcription factors was analyzed to further study the heterogeneity of T cell populations. With increased tumor PD-L1, expression of activation and cytotoxicity marker genes, including *IFNG*, *TNFSF9*, *TNFSF14*, *CSF2*, and *IL2*, were downregulated in both T_null_ and TCR-T_MART-1_ (Figure 4A), consistent with results of cytokine secretion assays (Figure 1F-G). In addition, in TCR-T_MART-1_ stimulated with PD-L1^high^, not only was the expression of anti-inflammatory cytokines, such as *IL10*, *IL13*, and *IL19*, upregulated, but the expression of pro-inflammatory cytokines, such as *IL12A*, *IL5*,* IL1A*, and* IL1b*, was also upregulated (Figure 4A). Moreover, anti-inflammatory *IL13* was generally expressed by C02, C06, C08, C11, C12, and C14 of T_null_ and TCR-T_MART-1_, while pro-inflammatory *IL5* was expressed by C11 and C06 of T_null_, and C11 and C08 of TCR-T_MART-1_ (Figure 4B). Little anti-inflammatory *IL10* and* IL19*, or pro-inflammatory *IL12A*, *IL1A*, and *IL1B* was expressed (Figure S4A). These results implied that both anti-inflammatory and pro-inflammatory cytokines could be upregulated by increased tumor PD-L1, and secreted by the same cluster. This further suggested that PD-L1 blockade might downregulate certain pro-inflammatory cytokines in some subsets of immune cells, leading to mixed efficacy results in clinical applications.

Chemokines were expressed higher in TCR-T_MART-1_ than T_null_ after antigen stimulation (Figure 4C). Since tumor PD-L1 may affect chemokine secretion and cell recruitment, the expression of *CCL4*, which was expressed similarly between T_null_ and TCR-T_MART-1_ targeting PD-L1^low^-, PD-L1^int^-, and PD-L1^high^-expressing tumor cells, was chosen for analysis. Differential expression patterns of *CCL4* and its receptor gene *CCR5* between T_null_ and TCR-T_MART-1_ were discovered (Figure 4D). In T_null_, the common clusters expressing *CCL4* were C11, C07, C12, and C14, except the additional cluster C02 targeting PD-L1^high^ tumor cells. CCL4 in T_null_ all recruited C07, C12 and C14 cell clusters by expressing *CCR5* (Figure 4D). The expression of *CCL4* and *CCR5* was more diverse in TCR-T_MART-1_ than in T_null_, and *CCL4* might recruit different cell clusters with the increasing tumor PD-L1 (Figure 4D). Specifically, *CCL4* expressed in TCR-T_MART-1_ targeting PD-L1^high^ tumors could recruit C05-Naïve-IL7R cells by expressing *CCR5*, which might result in a difference from other groups (Figure 4D). In conclusion, the chemokine expression and cell recruitment regulated by increased percentages of tumor PD-L1 might result in the mixed predictions seen in evaluating the efficacy of anti-PD-1/PD-L1 therapies using tumor PD-L1 as the sole biomarker.

Unique expression patterns of transcription factors (TFs) was also discovered in T_null_ and TCR-T_MART-1_ populations. The expression of *ZEB2*, *RBPJ*, *NFKB1*, *GATA3*, *IRF4*, and *STAT3*, which are important for T cell activation and differentiation 41 42, were higher in TCR-T_MART-1_ cultured with PD-L1^low^ tumors (Figure 4E). With increasing levels of tumor PD-L1, the expression of transcription factors for Th1, Th2, or M1 macrophages, including *NFKB1*, *GATA3*, *HIF1A*, *STAT4*, *TBX21*, *IRF8*, and *STAT1*, was downregulated (Figure 4F). However, the expression of transcription factors including *STAT3*, *IRF4*, *CEBPB*, and *AHR* for suppressive cell subsets such as regulatory T (Treg) cells and M2 macrophages was also decreased (Figure 4G). Therefore, increased tumor PD-L1 expression causes downregulation of both pro-inflammatory and anti-inflammatory transcription factors, which might partially explain the complicated effects brought on by increasing percentages of tumor PD-L1 and why standalone PD-L1inhibition might not always result in an efficacious anti-tumor response in patients.

### Increased tumor PD-L1 modulated both inhibitory and stimulatory checkpoint molecules in T cells

**Figure 5:**
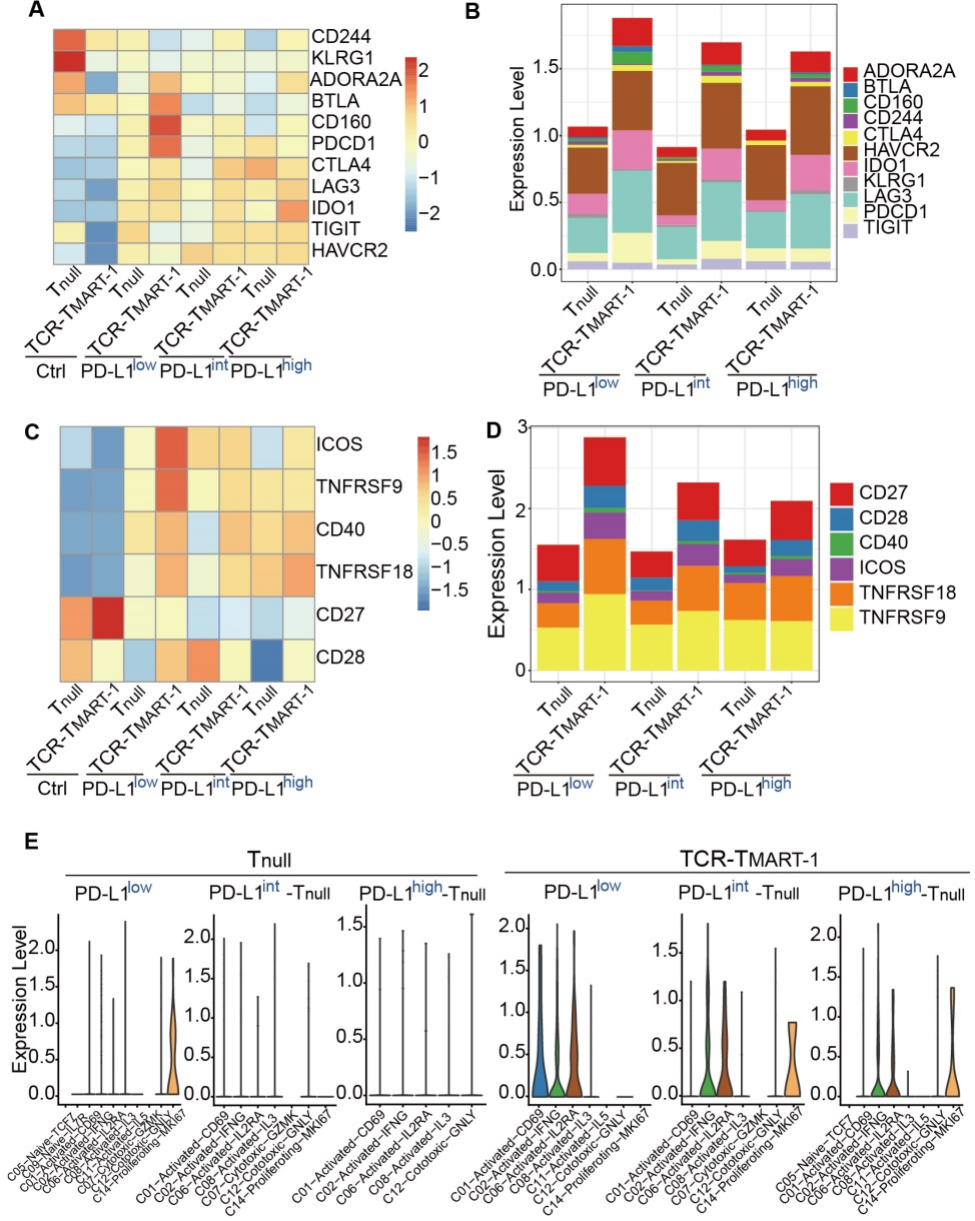
Increased tumor PD-L1 influenced both inhibitory and stimulatory checkpoint molecules in T cells.** (A)** Expression of inhibitory checkpoint molecules (ICMs) in T_null_ and TCR-T_MART-1_ with increased ratios of PD-L1^+^ tumor cells. **(B)** The bar plot shows the proportion distribution of cells expressing different ICMs in T_null_ and TCR-T_MART-1_ targeting different ratios of PD-L1^+^ tumor cells (cutoff: UMI of the gene > 0).** (C)** Expression of stimulatory checkpoint molecules (SCMs) in T_null_ and TCR-T_MART-1_. **(D)** The bar plot shows the proportion distribution of cells expressing different SCMs in T_null_ and TCR-T_MART-1_ targeting different ratios of PD-L1^+^ tumor cells (cutoff: UMI of the gene > 0). **(E)** The expression distribution of *ICOS* in cell clusters of T_null_ and TCR-T_MART-1_ responding to differential proportions of PD-L1^+^ tumor.

Blockade of PD-1 has been reported to lead to a compensatory upregulation of other checkpoint pathways 43. Thus, we analyzed whether increased tumor PD-L1 affected other checkpoint molecules. With increased tumor PD-L1, the expression of inhibitory checkpoint molecules (ICMs), including *ADORA2A*, *BTLA*, *CD160*, and *PDCD1*, was downregulated while the expression of *IDO1* was upregulated (Figure 5A). *ADORA2A* and *IDO1* were expressed more in TCR-T_MART-1_ than in T_null_, while *HAVCR2* and *LAG3* were generally expressed in T_null_ and TCR-T_MART-1_ (Figure 5B). Taken together, the contrary influences on different ICMs might lead to the heterogeneous response to tumor PD-L1, resulting in the mixed predictions on the efficacy of anti-PD-1/PD-L1 therapies based on tumor PD-L1 levels.

Simultaneously, the expression of stimulatory checkpoint molecules (SCMs) such as *ICOS* and *TNFRSF9* was highest in TCR-T_MART-1_ targeting PD-L1^low^ (Figure 5C), consistent with its greatest cytotoxicity. *CD27*, *TNFRSF18*, and *TNFRSF9* was generally expressed in T_null_ and TCR-T_MART-1_, while *CD28* and *ICOS* were expressed more in TCR-T_MART-1_ than in T_null_ (Figure 5D). The percentages of SCMs was not affected much by increased tumor PD-L1; however, the expression patterns of these SCMs in T cell clusters were modified (Figure 5E, Figure S5A). In T_null_, *ICOS* was only expressed in C14-Proliferating-MKI67 targeting PD-L1^low^, while in TCR-T_MART-1_, *ICOS* was commonly expressed in C02, C06, and additionally in C01 targeting PD-L1^low^ and C14 targeting PD-L1^int^ and PD-L1^high^ tumors (Figure 5F). Overall, percentages of ICMs and expression patterns of SCMs varied with the increased ratios of tumor PD-L1, which may contribute to the complicated responses caused by tumor PD-L1.

### Increased expression of tumor PD-L1 affected death of tumor cells and T cells with limited correlation

**Figure 6:**
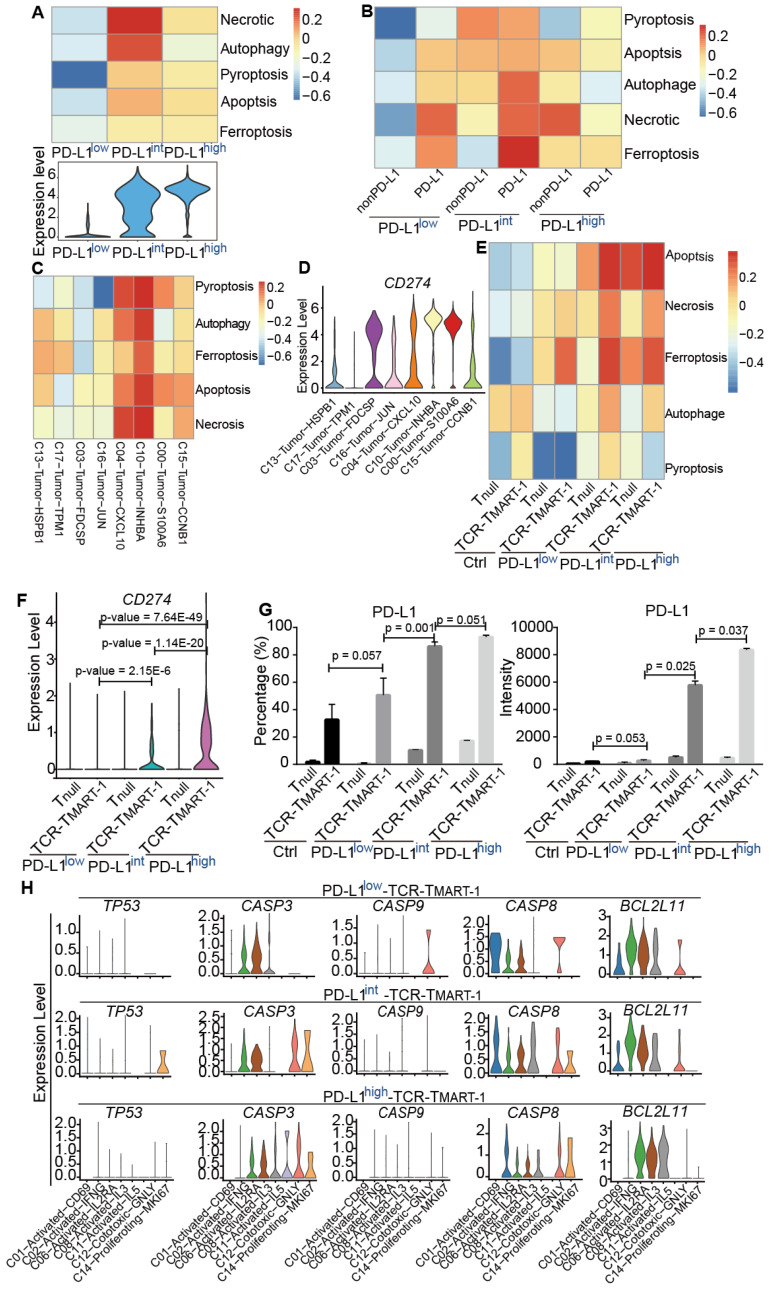
Increased expression of tumor PD-L1 affected death of tumor cells and T cells without direct correlation.** (A)** GSVA analysis of cell death pathways in tumor cells (top) and violin plot showing the expression level of PD-L1 in tumor cells (bottom). **(B)** GSVA analysis of cell death pathways in tumor cells either expressing PD-L1 or not. **(C)** GSVA analysis of cell death pathways in different tumor clusters. **(D)** The expression levels of PDL1 among cancer clusters.** (E)** GSVA analysis of cell death pathways in different subsets of T cells. **(F)** The expression of *CD274* in T_null_ and TCR-T_MART-1_ responding to differential proportions of PD-L1^+^ tumor. **(G)** The percentage (left) and intensity (right) of PD-L1 expression on tumor cells after incubation with MEL-526 cells for 24 h. **(H)** The expression distribution of apoptotic genes in T cell clusters of TCR-T_MART-1_ responding to differential proportions of PD-L1^+^ tumor.

To detect the impact of PD-L1 expression on tumor and immune cell death, gene sets of cell death pathways, including apoptosis, necrosis, autophagy, pyroptosis, and ferroptosis, were used for GSVA analysis. We first analyzed tumor cells after they were cocultured with T cells for 24 h. Cell death pathways, especially necrosis and autophagy, were most enriched in PD-L1^int^ (Figure 6A), suggesting a non-linear correlation between PD-L1 expression and tumor cell death at the transcriptional level. When tumor populations were separated into PD-L1-expressing or PD-L1-non-expressing (nonPD-L1) subsets, cell death pathways were most enriched in PD-L1-expressing cells of PD-L1^int^ tumors (Figure 6B). After further analysis, C10-Tumor-INHBA and C04-Tumor-CXCL10 had enriched the highest level of cell death signaling (Figure 6C), while the highest expression of *CD274* (encoding PD-L1) was in C10 and C00 (Figure 6D), implying a limited correlation between tumor *CD274* expression and tumor cell death.

To gain insight into whether immune cell death would be affected by tumor PD-L1, cell death pathways (Figure 6E) and gene expression (Figure S6A) were analyzed in T_null_ and TCR-T_MART-1_. Cell death pathways were more enriched in TCR-T_MART-1_ than in T_null_ in each group (Figure 6E). Amazingly, the expression of *CD274*/PD-L1 but not *PDCD1* (Figure S6B), was found to be gradually upregulated in TCR-T_MART-1_ targeting PD-L1^int^ and PD-L1^high^ tumors, both bioinformatically (Figure 6F) and experimentally (Figure 6G). Though the expression of *CD274*/PD-L1 was gradually elevated in TCR-T_MART-1_ with increased tumor PD-L1, there was limited correlation between *CD274* expression in T cells and T cell death (data not shown). The expression of apoptotic genes, including *TP53*, *CASP3*, *CASP9*, *CASP8*, and *BCL2L11*, was further analyzed in all the clusters of TCR-T_MART-1_. The expression of *CASP3* became more enriched in TCR-T_MART-1_ clusters with increased tumor PD-L1 (Figure 6H), implying that *CASP3* might play an important role in cell death enrichment. Overall, the non-direct correlation between PD-L1 expression and death of tumor cells or T cells demonstrated the complexity of the influences caused by tumor PD-L1.

### Consistency in the effect of tumor PD-L1 expression in melanoma patients

**Figure 7:**
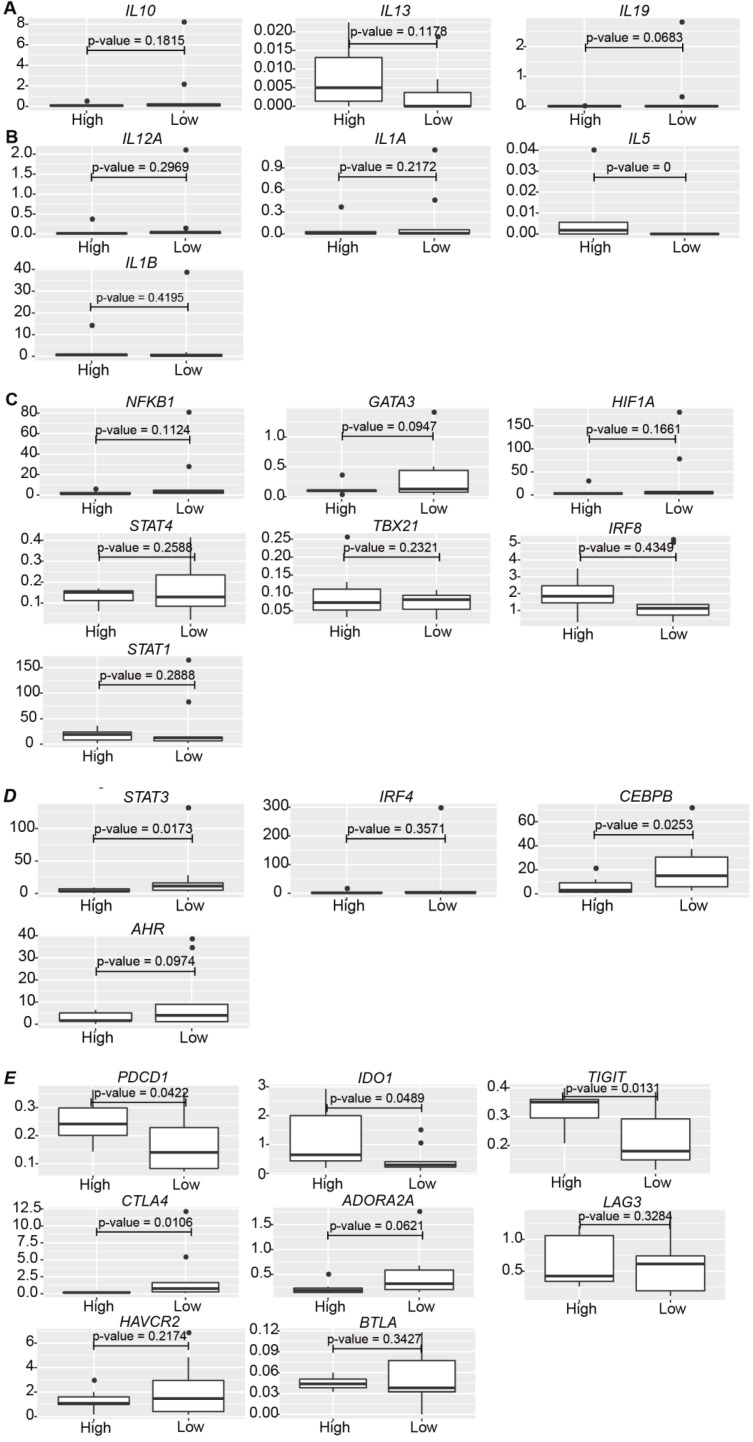
** The clinical relevance of the effect of PD-L1 expression on gene expression in melanoma patients. (A)** The expression of *IL10*, *IL13*, and* IL19* in PD-L1^low^ (n = 9) and PD-L1^high^ (n = 7) patients (Cutoff = 0.003). **(B)** The expression of *IL12A*, *IL1A*, *IL5*, and *IL1B* in PD-L1^low^ (n = 9) and PD-L1^high^ (n = 7) patients. **(C)** The expression of *NFKB1*, *GATA3*, *HIF1A*, *STAT4*, *TBX21*, *IRF8*, and *STAT1* in PD-L1^low^ (n = 9) and PD-L1^high^ (n = 7) patients. **(D)** The expression of *STAT3*, *IRF4*, *CEBPB*, and *AHR* in PD-L1^low^ (n = 9) and PD-L1^high^ (n = 7) patients. **(E)** The expression of *PDCD1*, *IDO1*, *TIGIT*, *CTLA4*, *ADORA2A*, *LAG3*, *HAVCR2*, and *BTLA* in PD-L1^low^ (n = 9) and PD-L1^high^ (n = 7) patients. The p value was all determined by permutation test.

Various levels of tumor PD-L1 are expressed in cancer patients. To determine whether tumor PD-L1 worked *in vivo* in the same way as that *in vitro*, various data sets were analyzed. However, it is hard to find proper single-cell sequencing data in melanoma patients, either due to the lack of sequencing information of malignant cells 44 or clinical treatment information 45, or the limitation of cell numbers from anti-PD-1/PD-L1-treated melanoma patients 46. ScRNA-seq profiles from one recently published paper 47 was evaluated (data not shown), but because of the low cell number of each sample (Table S1), the result was not reliable. We decided to examine the bulk data from melanoma patients 48 due to the lack of proper single-cell data. 16 samples remained after excluding patients with prior MAPK inhibitor treatment or patients who were on-treatment (Table S2). HSPB1 and KRT18 were the tumor marker genes investigated, and patients were classified into PD-L1^low^ and PD-L1^high^ groups based on the relative expression of *CD274* (Cutoff = 0.003, described in Methods) (Table S2). No correlation was discovered between the relative expression of *CD274* and the patient response to Pembrolizumab treatment (odds ratio = 0.9413123, p value = 1). The expression of anti-inflammatory *IL13* and pro-inflammatory *IL5* was higher in PD-L1^high^ patients than PD-L1^low^ patients (Figure 7A, 7B), consistent with our *in vitro* results (Figure 4A), while there was no dramatic difference in the expression of *IL10*, *IL19* (Figure 7A), *IL12A*, *IL1A*, and *IL1B* (Figure 7B) between PD-L1^high^ and PD-L1^low^ patients.

No significant change was found in the expression of transcription regulators for the pro-inflammatory immune cells, such as *NFKB1*, *GATA3*, *HIF1A*, etc. between PD-L1^high^ and PD-L1^low^ patients (Figure 7C). However, in line with TCR-T_MART-1_ responding to tumor PD-L1 (Figure 4G), CD8^+^ T cells in PD-L1^low^ patients had higher expression of *STAT3* and *CEBPB* (Figure 7D), which are transcription factors for immune-suppressive cell subsets. Immune checkpoint molecules were also differently expressed in PD-L1^high^ and PD-L1^low^ patients as well (Figure 7E). The expression of *PDCD1*, *IDO1*, and *TIGIT* was much higher in PD-L1^high^ patients than PD-L1^low^ patients, while *CTLA4* and *ADORA2A* had a contrary expression pattern (Figure 7E). However, there was no dramatic difference in the expression of *LAG3*, *HAVCR2*, and *BTLA* between PD-L1^high^ and PD-L1^low^ patients. In summary, consistent with our *in vitro* results, higher levels of tumor PD-L1 in melanoma patients was not only representative of higher anti-inflammatory characteristics, such as higher expression of *IL13*, *PDCD1*, *IDO1*, and *TIGIT*, but also brought up pro-inflammatory features, such as higher expression of *IL5*, lower expression of *STAT3*, *CEBPB*, and *AHR*, and suppressive immune checkpoint molecules *CTLA4* and *ADORA2A*. Thus, it is likely that the blockade of tumor PD-L1 would not only suppress the anti-inflammatory characteristics but also the pro-inflammatory features triggered by tumor PD-L1, causing a complex situation in predicting the efficacy of PD-1/PD-L1 blockade based only on the expression level of tumor PD-L1.

## Discussion

It has been well documented that the efficacy of CPI for cancer treatment is affected by PD-L1 expression levels, its interaction with tumors, and the relative amounts of PD-1 9. However, the molecular mechanism by which different levels of tumor PD-L1 expression affects the therapeutic efficacy of TCR-T cell therapy remains unclear.

Few studies have reported the effects of PD-L1 expression levels on TCR-T cell function. Our study provides an insight on TCR-T cell response to different proportions of tumor cells expressing PD-L1 at the single-cell level. The results of cell-based assays revealed that higher proportions of PD-L1^+^ tumor cells more strongly inhibited T-cell function than lower proportions of PD-L1^+^ tumor cells (Figure 1D-G). More importantly, single-cell transcriptome profiling demonstrated a comprehensive landscape of the modifications caused by the differential expression of tumor PD-L1, including cluster features (Figure 2), responses of clusters and T cell populations (Figure 3), expression of cytokines, chemokines, transcription factors (Figure 4), and checkpoint molecules (Figure 5), as well as tumor and immune cell death (Figure 6).

Activated or cytotoxic cells from different clusters reacted differently to the increased ratios of tumor PD-L1 (Figure 3B, C02&C06 vs. C01&C11&C12), indicating that tumor PD-L1 might promote certain, but not all, T cell subsets that are capable of killing tumor cells. This discovery indicated that blocking PD-L1 might decrease the percentage of some activated or cytotoxic T cell subpopulations and this might be one of the reasons why the expression of tumor PD-L1 may not accurately predict the outcome of anti-PD-1/PD-L1 therapies.

TCR-T_MART-1_ were more vulnerable than T_null_ when targeting increasing proportions of PD-L1-bearing tumor cells (Figure 3B, 3E). This indicates that TCR-T therapy could be co-administrated with PD-L1/PD-1 interference to obtain better anti-tumor efficacies. Clinical trials with TCR-T cells armed with a PD-1 antagonist are ongoing (NCT04139057, NCT03578406). The result also implies that TCR-T cells will benefit from elimination of their *PDCD1*, perhaps by using CRISPR-based approaches, to protect themselves against PD-L1-mediated inhibition 49.

In addition to anti-inflammatory cytokines, such as *IL13*, the expression of pro-inflammatory cytokines, such as *IL5*, was upregulated in TCR-T_MART-1_ targeting PD-L1^high^ (Figure 4A-B). The result implied that the inhibition of PD-L1 might downregulate certain pro-inflammatory cytokines, which might be one reason why tumor PD-L1 is not a perfect predictor for the efficacy of PD-1/PD-L1 blockade. Moreover, the finding that combined blockade of PD-L1 and IL10 further enhanced T-cell immunity 50, 51 suggests that IL13 may also have the potential to be targeted together with PD-1/PD-L1 to increase anti-tumor function. In addition to cytokines, the expression of chemokines and cell recruitment was modified by increased tumor PD-L1 as well, demonstrating the complexity of the effects caused by tumor PD-L1.

Transcription factors play critical roles in immunity, such as TBX21 (T-bet), which activates transcription of IFN- gene and enhances Th1 cell development 52. After stimulation by increased expression of tumor PD-L1, the expression of transcription factors responsible for the development of pro-inflammatory and anti-inflammatory cell populations was downregulated (Figure 4E-F). This finding suggested that PD-L1 blockade in clinical applications might upregulate some specific anti-inflammatory cell populations, resulting in the contradicting predictions for the effectiveness of PD-1/PD-L1 blockade by tumor PD-L1. Increased expression of tumor PD-L1 regulated different ICMs contrarily (Figure 5B) and modulated the expression patterns of SCMs (Figure 5F), which further promoted the complexity of the roles of tumor PD-L1.

Various cell death pathways were involved in tumor and T cell death (Figure 6). The death of neither tumor cells nor T cells correlated well with the expression level of PD-L1, though the expression of PD-L1 on T cells was dose-dependently increased by the elevation of tumor PD-L1 expression (Figure 6F-G). PD-L1 was reported to be expressed on T cells 53-55, and a recent research found that PD-L1 is up-modulated on T cells in cancers responding to antigen presentation, which suppresses neighboring macrophages and effector T cells and promotes self-tolerance 56. Thus, the upregulated expression of PD-L1 on T cells might play a suppressive role in T cell function and anti-tumor activity.

There are some limitations in this work. PD-L1 was overexpressed in one melanoma cell line in our study, which may be different from human primary melanoma due to a more complicated microenvironment. Due to the lack of proper single-cell sequencing data from melanoma patients, we could only partly confirm our findings using clinical bulk data, while the prognostic role of different populations of CD8^+^ T cells could not be defined based on the bulk data from melanoma patients treated with anti-PD1/PD-L1.

## Conclusions

Cell-based cytotoxicity and cytokine secretion assays in conjunction with scRNA-seq were applied to interrogate MART-1-specific transgenic T cells upon antigen-specific stimulation with different ratios of tumor PD-L1. This study provides the first comprehensive illustration of tumor PD-L1 influences on TCR-T cell function at the single-cell level, and reveals new findings regarding the heterogenous effects caused by increased tumor PD-L1 on TCR-T cells. It provides valuable information about why the PD-L1 blockade might promote not only pro-inflammatory responses, but also anti-inflammatory responses at the transcriptome level.

## Supplementary Material

Supplementary figures.

Supplementary table 1.

Supplementary table 2.

## Abbreviations

PD-L1: programmed cell death-ligand-1
PD-1: programmed death-1
IFN-γ: interferon-γ
TILs: tumor infiltrating lymphocytes
NSCLC: non-small-cell lung cancer
APC: antigen presenting cell
CPI: checkpoint inhibitor
scRNA-seq: single-cell mRNA sequencing
PBMC: peripheral blood mononuclear cells
DMSO: dimethyl sulfoxide
PE: phycoerythrin
CFSE: Carboxyfluorescein succinicmidyl ester
CBA: cytometric bead array
SD: standard deviation
UMI: unique molecular identifier
GO: Gene Ontology
FACS: fluorescence-activated cell sorting
E:T: effector:target
OE: overexpressed
WT: wild-type
DEG: differentially expressed genes
Ctrl: control
GSVA: gene set variation analysis
TF: transcription factor
ER: endoplasmic reticulum
ICM: inhibitory checkpoint molecule
SCM: stimulatory checkpoint molecule
COVID-19: coronavirus disease 2019
SARS-CoV-2: severe acute respiratory syndrome coronavirus 2
mDC: myeloid dendritic cell
pDC: plasmacytoid dendritic cell
IL-6: interleukin 6
BALF: bronchoalveolar lavage fluid
ICI: immune checkpoint inhibitors
