# Supplementary material for: Single-cell transcriptome analysis of the heterogeneous effects of differential expression of tumor PD-L1 on responding TCR-T cells
Source: Theranostics. 2021 Mar 5;11(10):4957–74. doi: 10.7150/thno.55075 (PMC7978322; doi:10.7150/thno.55075)
Supplement: Supplementary file 1 — Supplementary figures. [file thnov11p4957s1.pdf]

Supplementary figures

Figure S1. $TCR_{MART\text{-}1}$ construction. **(A)** Schematic design of $TCR_{MART\text{-}1.}$ **(B)** Expression of $TCR_{MART\text{-}1}$ on $CD8^+$ T cells transfected by lentivirus before (middle) and after (right) cell sorting. **(C)** Killing of T2 cells by T_{null} and TCR-$T_{MART\text{-}1}$ after co-incubation for 6 h at E:T ratio of 1:1. **(D)** PD-L1 was over expressed in MEL-526 cells.

Figure S2. Cell clustering and tumor clusters. (**A**) UMAP visualization showing clustering of single cells among four experiment designs, colored by clusters. **(B)** Violin plot showing the expression of $TCR_{MART\text{-}1}$ in different cell clusters. (**C**) Heatmap of tumor cell clusters with unique signature genes. (**D**) The point plot shows the order of T cells along pseudotime in a two-dimensional state-space defined by Monocle2.

Figure S3. Characterization of T cell clusters, and difference between T_{null} and TCR-$T_{MART\text{-}1}$. (**A**) The expression of top 10 DEGs in C01, C02, C06, C11 and C12. (**B**) The violin plots show the expression distribution of cytokine and cytotoxicity genes in the five clusters **(C)** Differentially expressed genes in T_{null} responding to differential proportion of PD-$L1^+$ tumor. **(D)** The bubble plot showing the top 5 pathways in T_{null} and TCR-$T_{MART\text{-}1}$ targeting $PDL1^{low}$, $PDL1^{int}$, $PDL1^{high}$ tumor cells.

Figure S4. The expression of interleukins in T cell clusters. **(A)** The expression distribution of *IL10*, *IL19*, *IL12A*, *IL1A*, and *IL1B* in T cell clusters of T_{null} and TCR-$T_{MART\text{-}1}$ responding to PD-$L1^{high}$.

Figure S5. Expression of checkpoint molecules in clusters of T_{null} and TCR-$T_{MART\text{-}1}$. **(A)** The expression distribution of *CD28*, *CD27*, *TNFRSF18*, and *TNFRSF9* in cell clusters of T_{null} and TCR-$T_{MART\text{-}1}$ responding to differential proportion of PD-L1^{+} tumor.

Figure S6. Enrichment of cell death signaling in T cells and expression of *PDCD1* and *CD274*. **(A)** Heatmap of gene members from different cell death pathways. **(B)** The violin plot showing the expression level of *PDCD1* in T_{null} and TCR-$T_{MART\text{-}1}$ responding to differential proportion of PD-L1^{+} tumor.

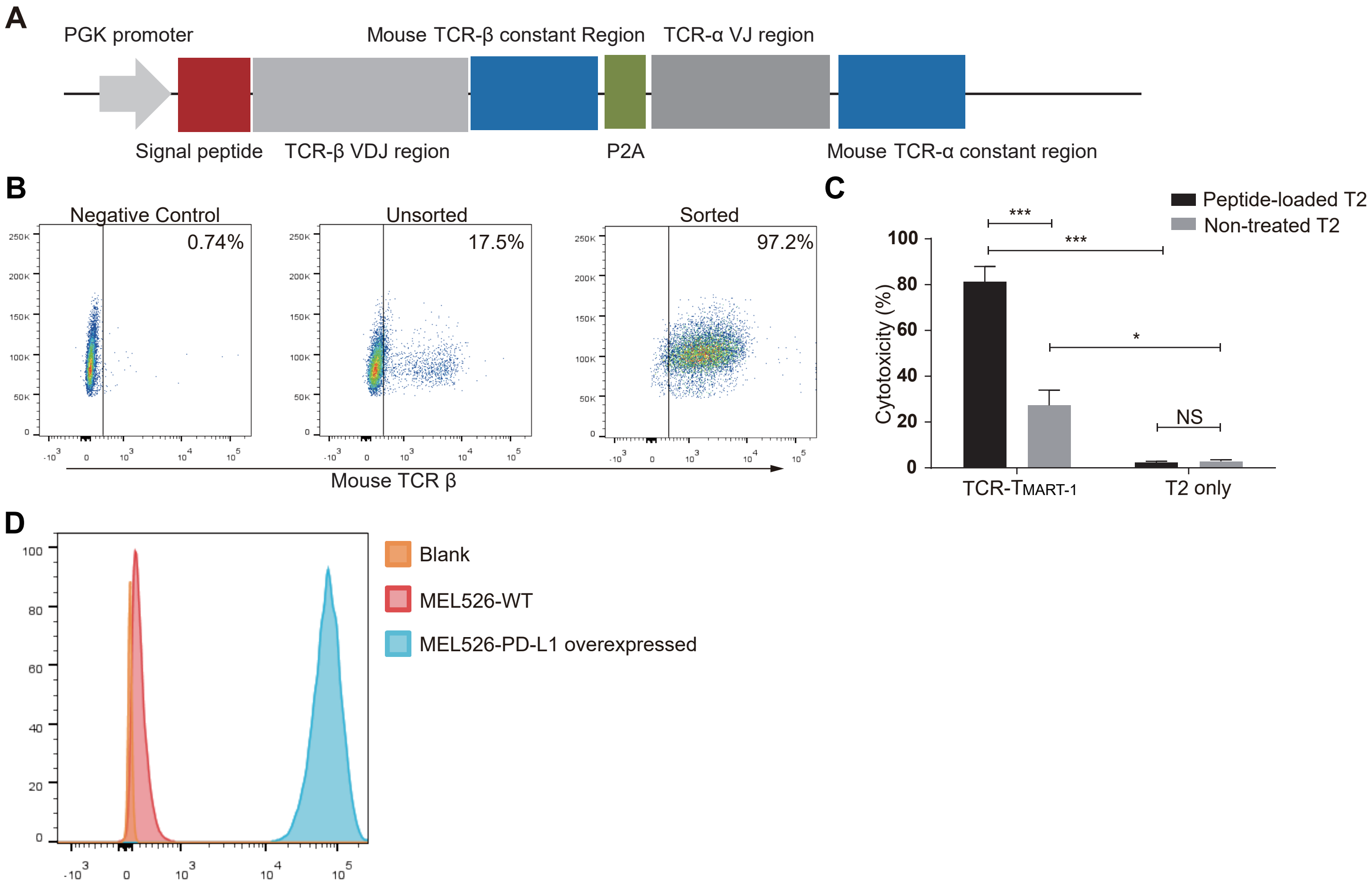
A
PGK promoter
Mouse TCR-β constant Region
TCR-α VJ region
Signal peptide
TCR-β VDJ region
P2A
Mouse TCR-α constant region
B
Negative Control
0.74%
Unsorted
17.5%
Sorted
97.2%
Mouse TCR β
C
Peptide-loaded T2
Non-treated T2
Cytotoxicity (%)
***
***
*
NS
TCR-TMART-1
T2 only
D
Blank
MEL526-WT
MEL526-PD-L1 overexpressed

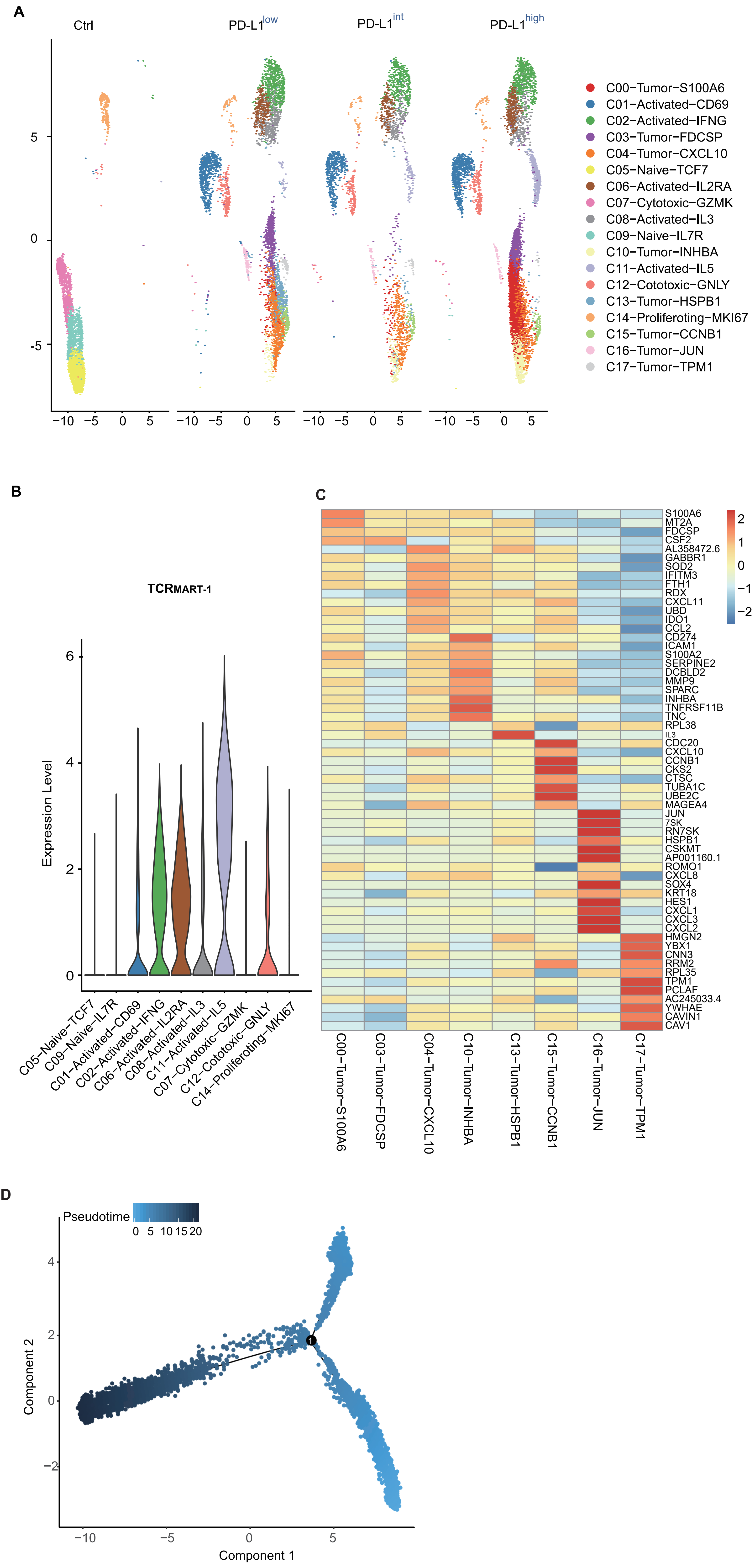
A
Ctrl
PD-L1low
PD-L1int
PD-L1high
C00−Tumor−S100A6
C01−Activated−CD69
C02−Activated−IFNG
C03−Tumor−FDCSP
C04−Tumor−CXCL10
C05−Naive−TCF7
C06−Activated−IL2RA
C07−Cytotoxic−GZMK
C08−Activated−IL3
C09−Naive−IL7R
C10−Tumor−INHBA
C11−Activated−IL5
C12−Cototoxic−GNLY
C13−Tumor−HSPB1
C14−Proliferoting−MKI67
C15−Tumor−CCNB1
C16−Tumor−JUN
C17−Tumor−TPM1
B
TCRMART-1
Expression Level
C05−Naive−TCF7
C09−Naive−IL7R
C01−Activated−CD69
C02−Activated−IFNG
C06−Activated−IL2RA
C08−Activated−IL3
C11−Activated−IL5
C07−Cytotoxic−GZMK
C12−Cototoxic−GNLY
C14−Proliferoting−MKI67
C
S100A6
MT2A
FDCSP
CSF2
AL358472.6
GABBR1
SOD2
IFITM3
FTH1
RDX
CXCL11
UBD
IDO1
CCL2
CD274
ICAM1
S100A2
SERPINE2
DCBLD2
MMP9
SPARC
INHBA
TNFRSF11B
TNC
RPL38
IL3
CDC20
CXCL10
CCNB1
CKS2
CTSC
TUBA1C
UBE2C
MAGEA4
JUN
7SK
RN7SK
HSPB1
CSKMT
AP001160.1
ROMO1
CXCL8
SOX4
KRT18
HES1
CXCL1
CXCL3
CXCL2
HMGN2
YBX1
CNN3
RRM2
RPL35
TPM1
PCLAF
AC245033.4
YWHAE
CAVIN1
CAV1
C00−Tumor−S100A6
C03−Tumor−FDCSP
C04−Tumor−CXCL10
C10−Tumor−INHBA
C13−Tumor−HSPB1
C15−Tumor−CCNB1
C16−Tumor−JUN
C17−Tumor−TPM1
D
Pseudotime
Component 2
Component 1

Supplementary Fig. 3

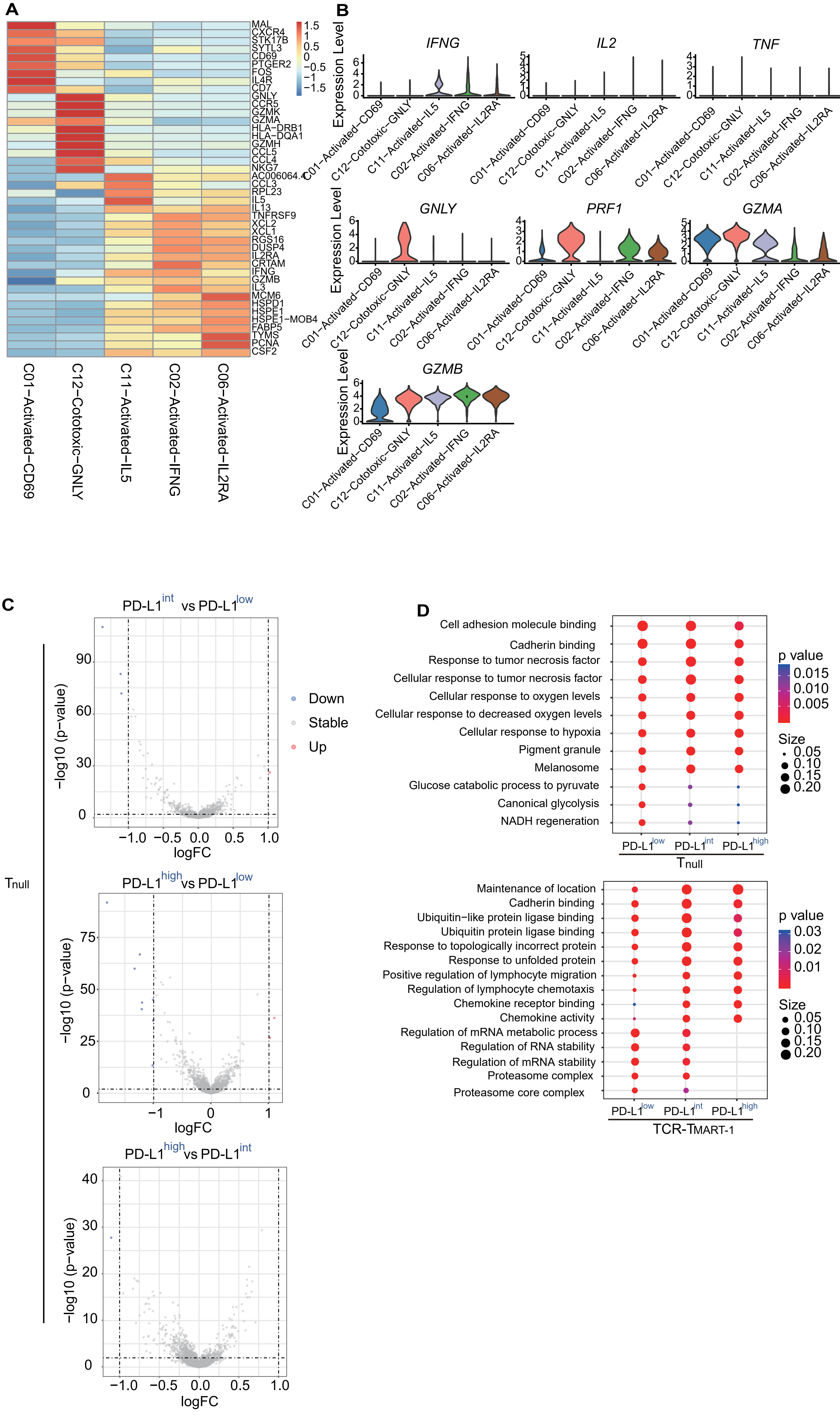

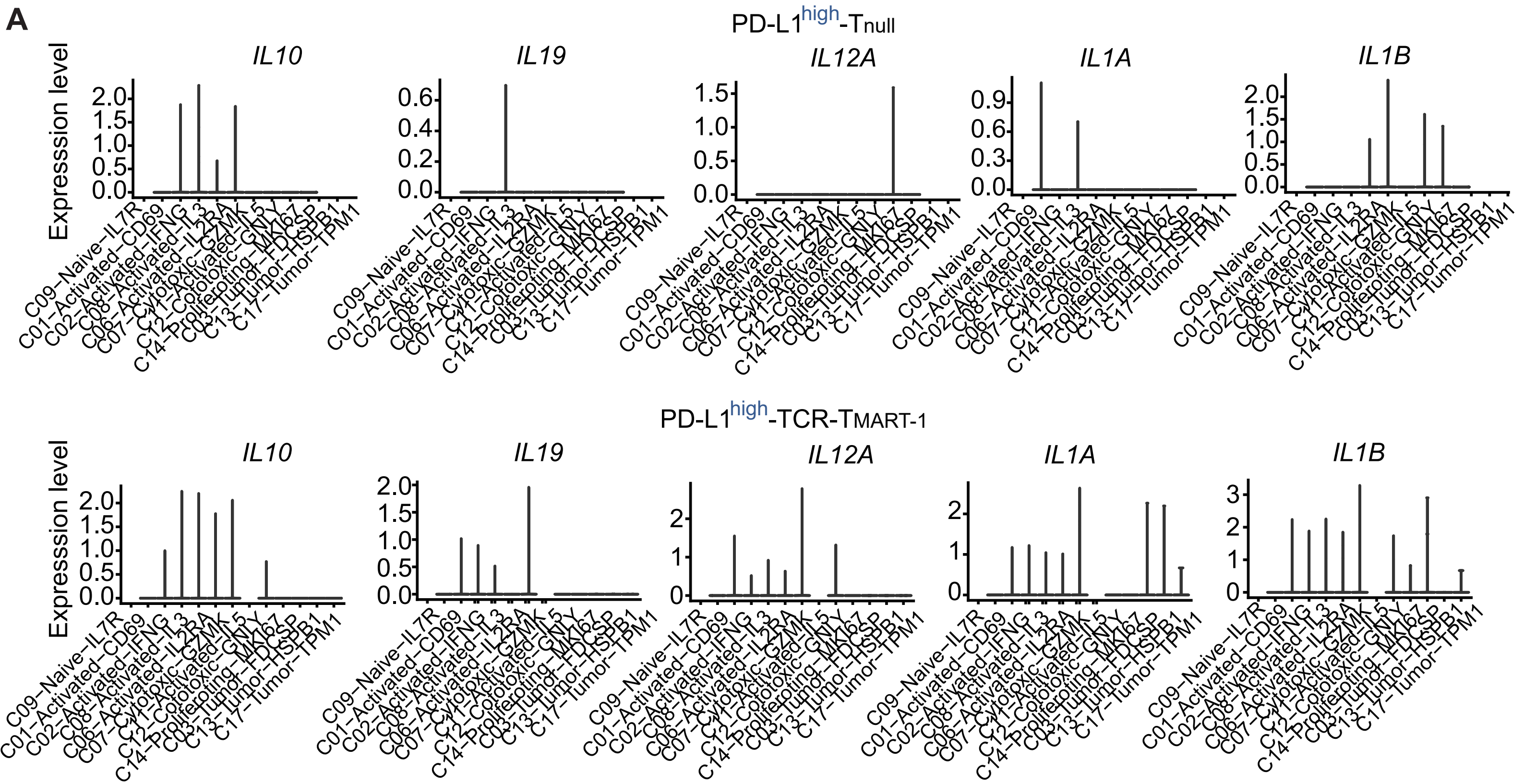
A
PD-L1high-Tnull
PD-L1high-TCR-TMART-1
IL10
IL19
IL12A
IL1A
IL1B
Expression level
C09–Naive–IL7R
C01–Activated–CD69
C02–Activated–IFNG
C08–Activated–IL3
C06–Activated–IL2RA
C07–Cytotoxic–GZMK
C11–Activated–IL5
C12–Cytotoxic–GNLY
C14–Proliferating–MKI67
C03–Tumor–FDCSP
C13–Tumor–HSPB1
C17–Tumor–TPM1

A

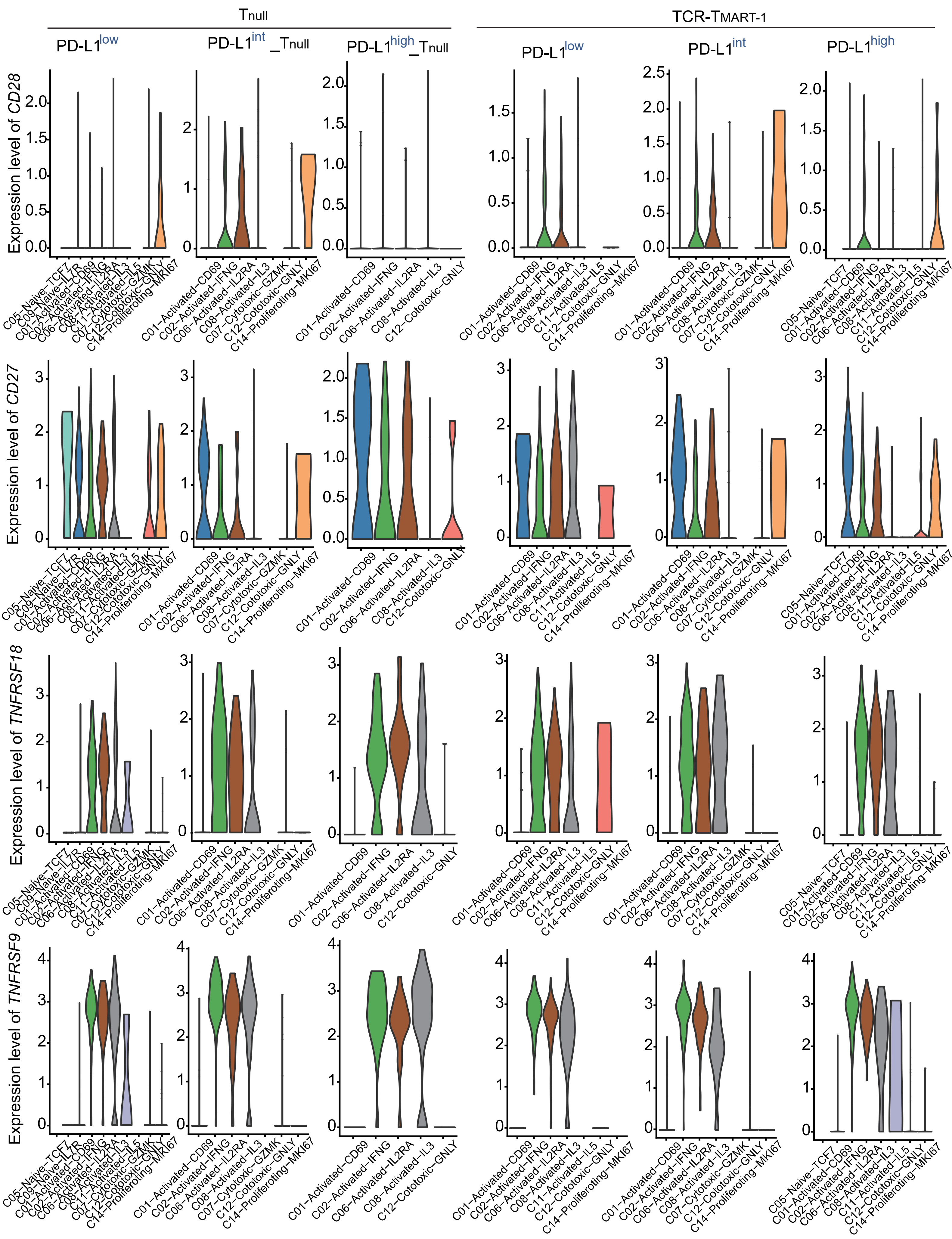

Tnull
TCR-TMART-1
PD-L1low
PD-L1int_Tnull
PD-L1high_Tnull
PD-L1low
PD-L1int
PD-L1high
Expression level of CD28
Expression level of CD27
Expression level of TNFRSF18
Expression level of TNFRSF9
C05–Naive–TCF7
C09–Naive–IL7R
C01–Activated–CD69
C02–Activated–IFNG
C06–Activated–IL2RA
C08–Activated–IL3
C11–Activated–IL5
C07–Cytotoxic–GZMK
C12–Cototoxic–GNLY
C14–Proliferoting–MKI67

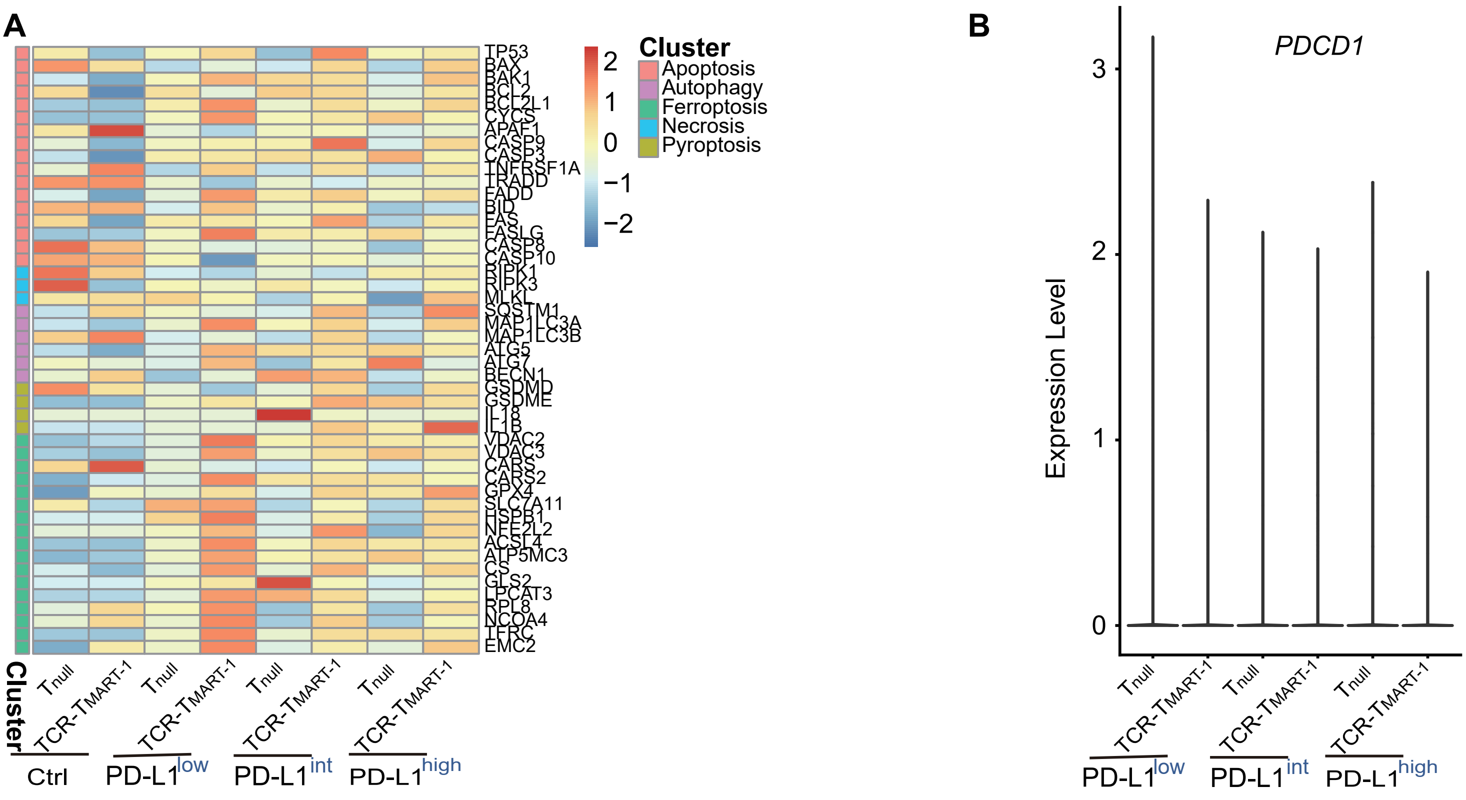
A
B
TP53
BAX
BAK1
BCL2
BCL2L1
CYCS
APAF1
CASP9
CASP3
TNFRSF1A
TRADD
FADD
BID
FAS
FASLG
CASP8
CASP10
RIPK1
RIPK3
MLKL
SQSTM1
MAP1LC3A
MAP1LC3B
ATG5
ATG7
BECN1
GSDMD
GSDME
IL18
IL1B
VDAC2
VDAC3
CARS
CARS2
GPX4
SLC7A11
HSPB1
NFE2L2
ACSL4
ATP5MC3
CS
GLS2
LPCAT3
RPL8
NCOA4
TFRC
EMC2
2
1
0
−1
−2
Cluster
Apoptosis
Autophagy
Ferroptosis
Necrosis
Pyroptosis
Cluster
Tnull
TCR-TMART-1
Ctrl
PD-L1low
PD-L1int
PD-L1high
PDCD1
Expression Level
3
2
1
0
